# Supplementary figures and images for: Order matters: How covert value updating during sequential option sampling shapes economic preference
Source: PLoS Comput Biol. 2020 Aug 11;16(8):e1007920. doi: 10.1371/journal.pcbi.1007920 (PMC7418959; doi:10.1371/journal.pcbi.1007920)

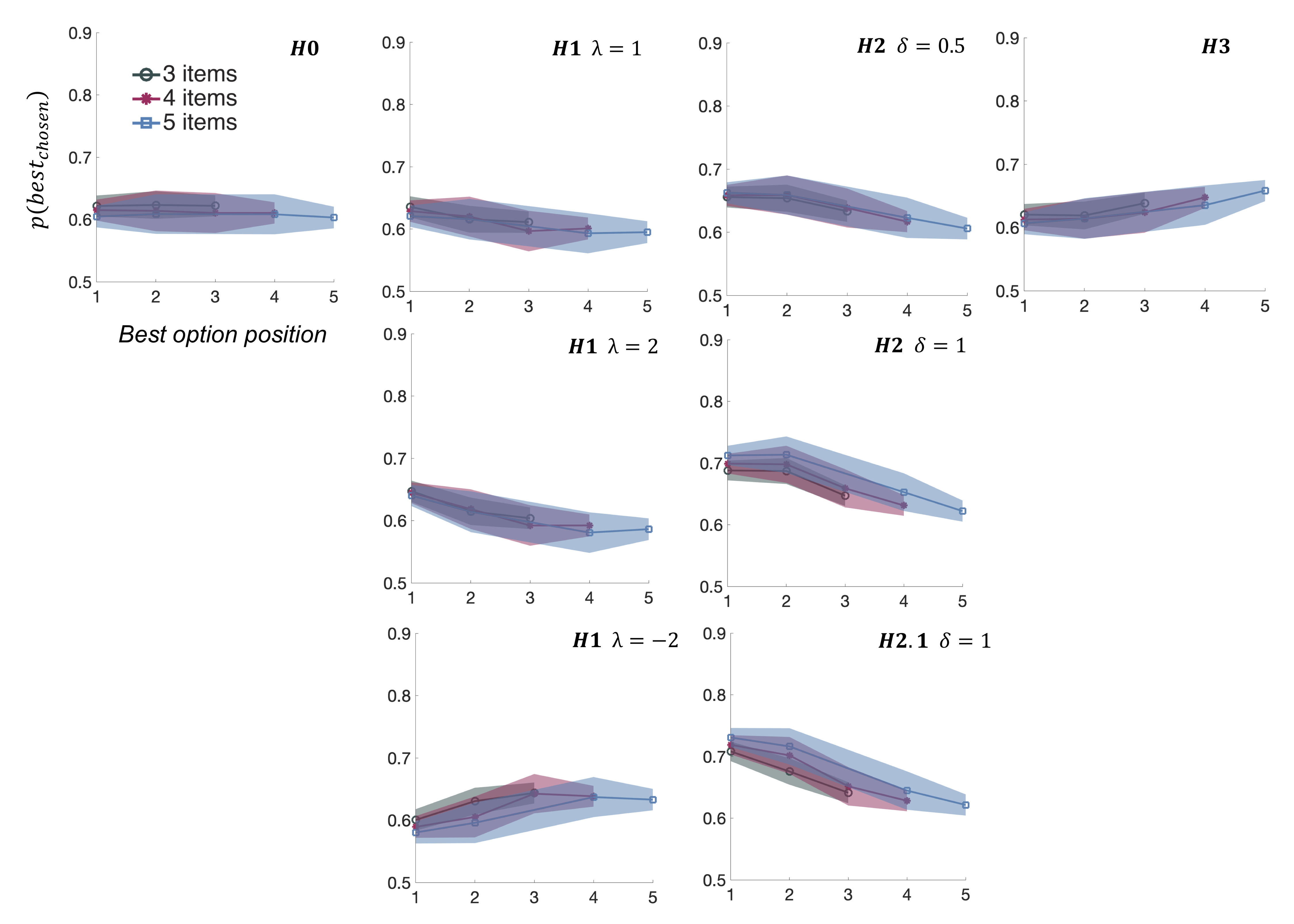

Supplement: S1 Fig — Graphs show simulated probability of choosing the best option under the experimental setup of Exp 2 and 3, depending on the serial position of the best option in the sampling sequence (x-axis). Each of the plots is an average over 200 simulated datasets of 30 subjects implementing the corresponding model, for various (color-coded) number of options. Shaded areas indicate the average inter-participant SEM, across all datasets. Values of λ and δ are indicated on the plots. The inverse temperature parameter was fixed to β = 0.10, which corresponds to the posterior estimates of the best model (H2.1) fitted to choice data in Exp 2. The simulations show that predictions about the link between P(best) and its serial position are similar to those made under Exp 1 settings. In particular, only H1 (with a positive bias) and H2 (including H2.1) predict a decreased choice rate when the best option is presented later in the sequence. (TIF) [file pcbi.1007920.s002.tif]

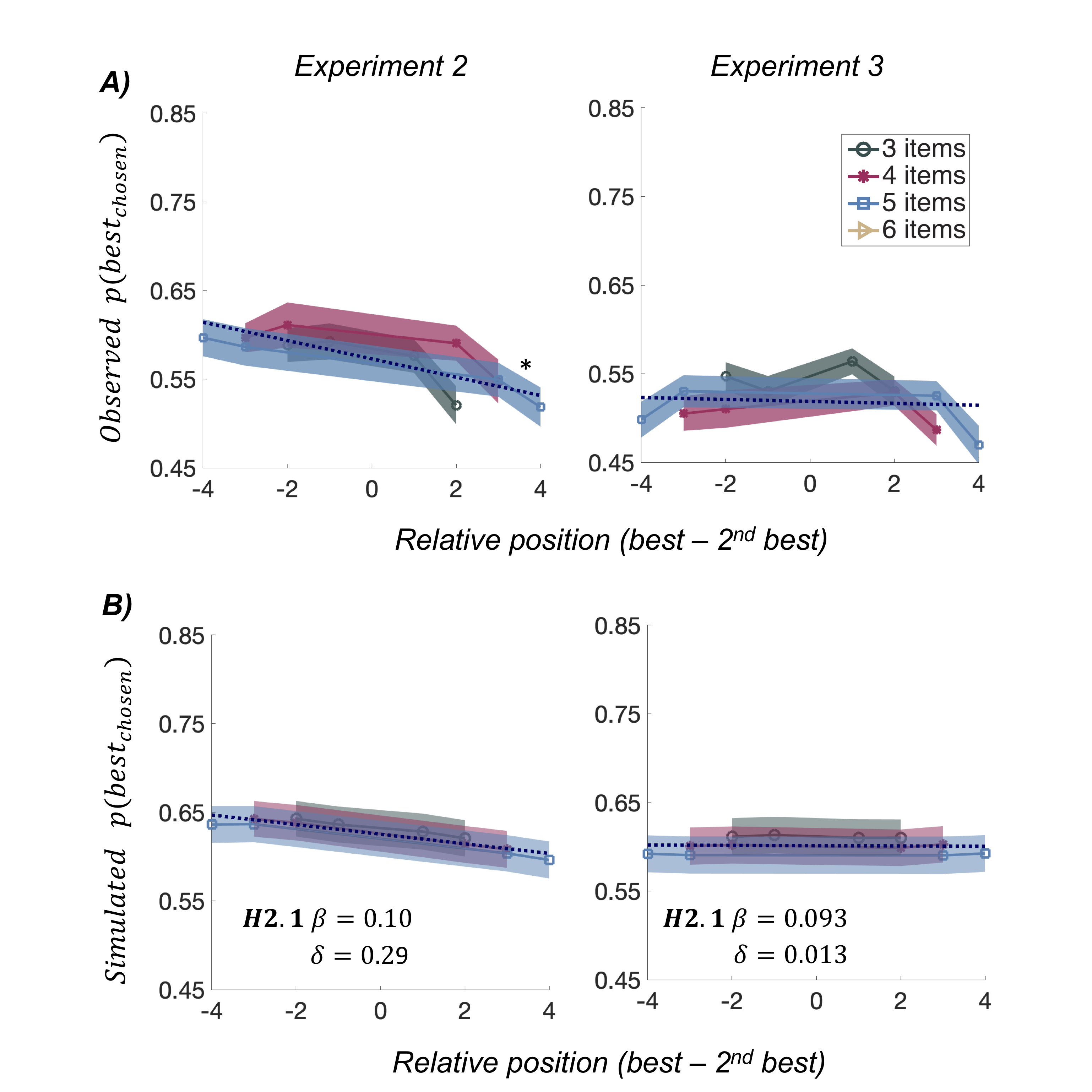

Supplement: S2 Fig — (A) The upper graphs show the observed probability of choosing the best option, as a function of its serial position relative to that of the second-best option. A positive relative position means that the best was presented after the second-best option. Shaded areas indicate inter-participant SEM. Dotted lines show linear regression fit across all trials (with different numbers of options). Stars denote significance of t-test comparing regression slopes to zero. * p<0.05, ** p<0.01. (B) The bottom graphs show the simulated probability of choosing the best option, as a function of its serial position relative to that of the second-best option. Choice behavior in each condition was simulated using the best-fitting model with the posterior means for free parameters (see values of the inverse temperature β and bonus δ indicated on the plots). Each of the plots is an average over 200 simulated datasets of 30 subjects implementing the corresponding model, for various (color-coded) number of options. Shaded areas indicate the average inter-participant SEM. across all datasets. (TIF) [file pcbi.1007920.s003.tif]

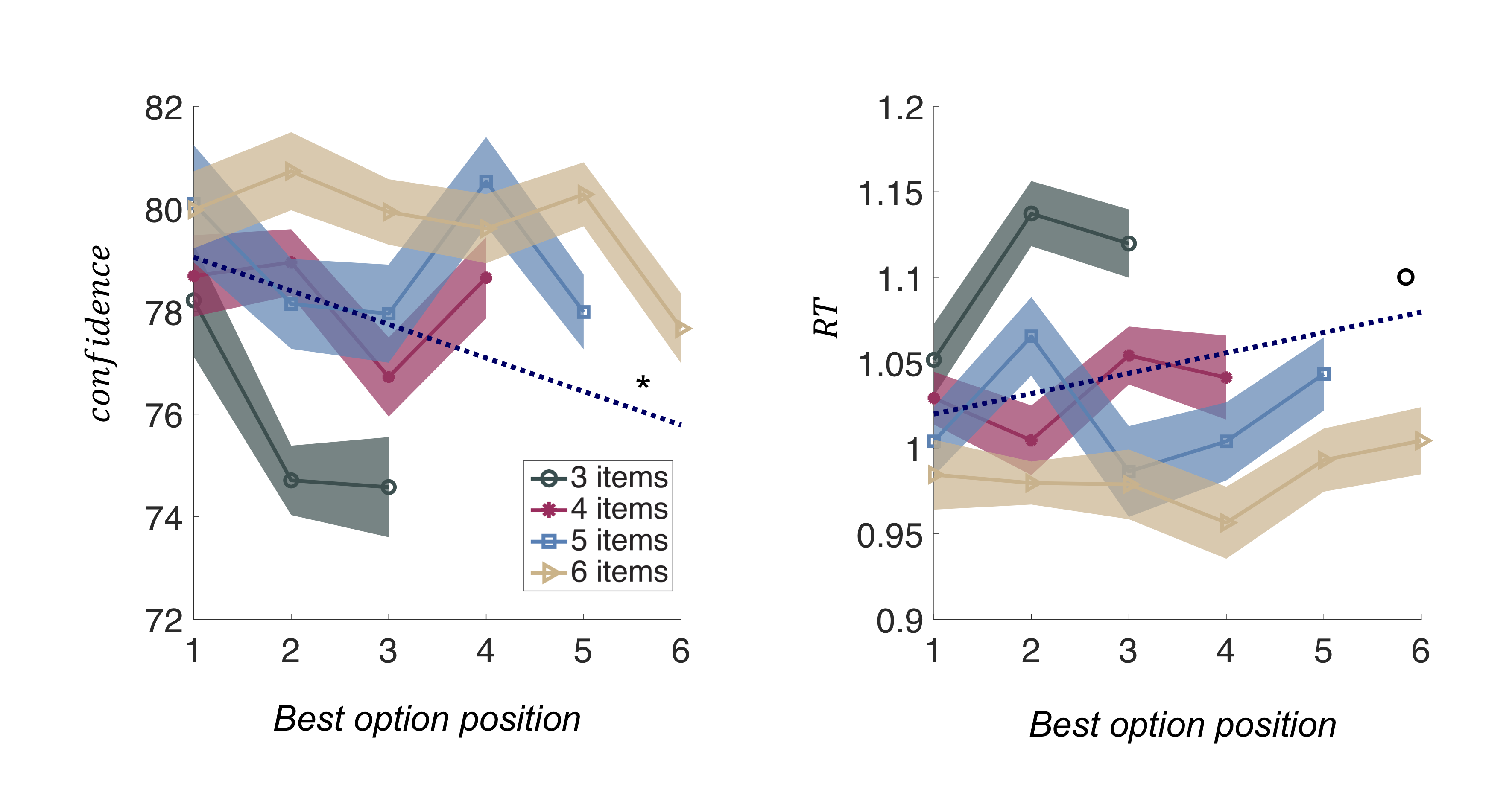

Supplement: S3 Fig — Graphs show the observed confidence rating (between 0 and 100) and response time (in seconds), as a function of the serial position of the best option (in Exp 1), for different (color-coded) number of options, averaged over trials in which the best option was chosen. Shaded areas indicate inter-participant SEM. Dotted lines show linear regression fit across trials (with different numbers of options). Star and circle denote significance or borderline significance of t-test comparing regression slopes to zero: * p<0.05,° p<0.1. (TIF) [file pcbi.1007920.s004.tif]

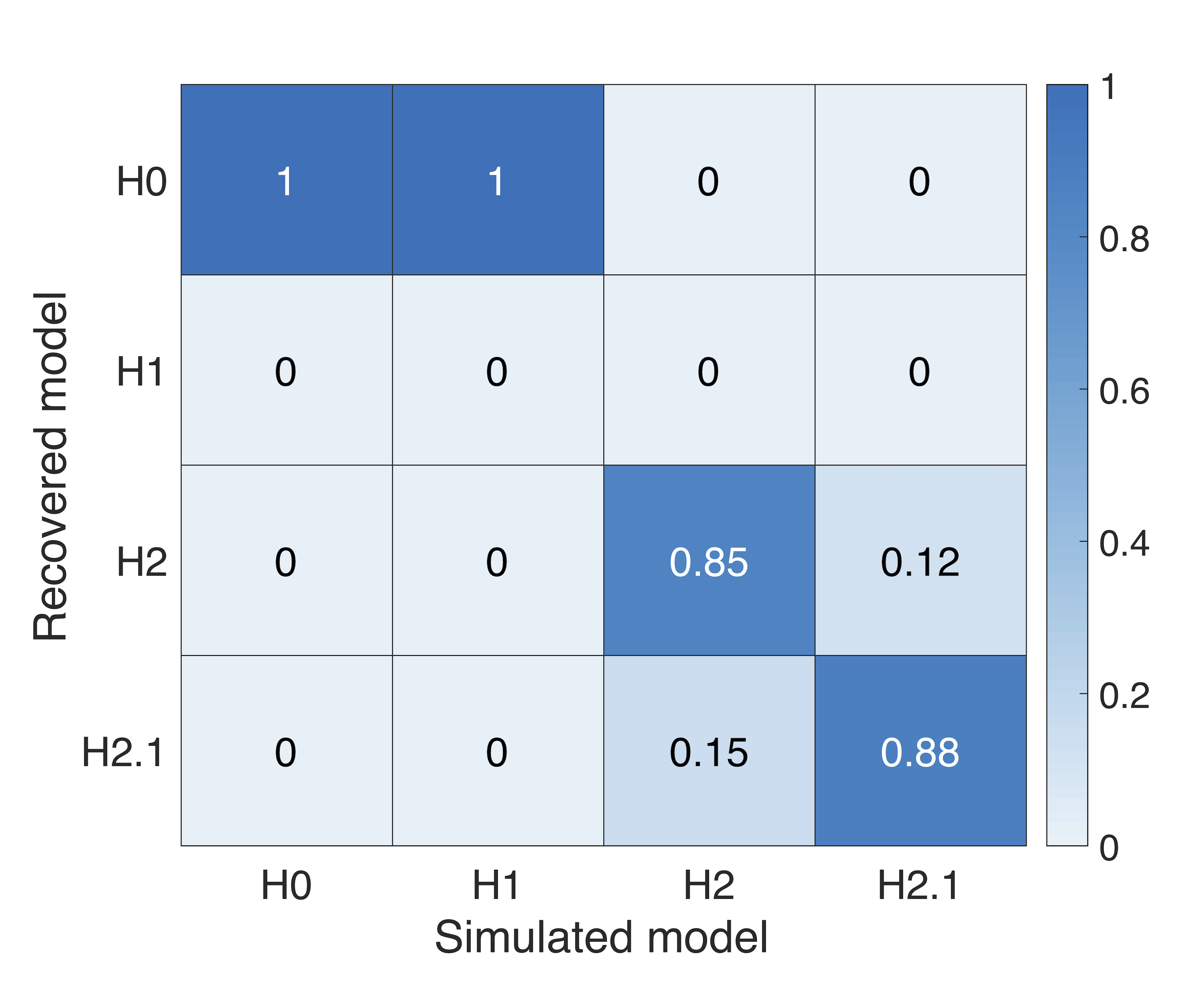

Supplement: S4 Fig — Choice data have been simulated using the likeability ratings and the posterior parameters of participants in Exp 1. Recovery rate has been established on the basis of 50 simulations, each including a group of 30 random participants. Cells of the confusion matrix indicate the rate at which the model in row wins Bayesian comparison when the model in column was simulated. Bayesian comparison was applied to simulated data at the group level, between the four considered models, following the exact same procedure applied to observed data. Only winning models with exceedance probability > 0.95 have been included in the count. Note that when H2.1 model wins the comparison, the simulated model is either H2 or H2.1. (TIF) [file pcbi.1007920.s005.tif]

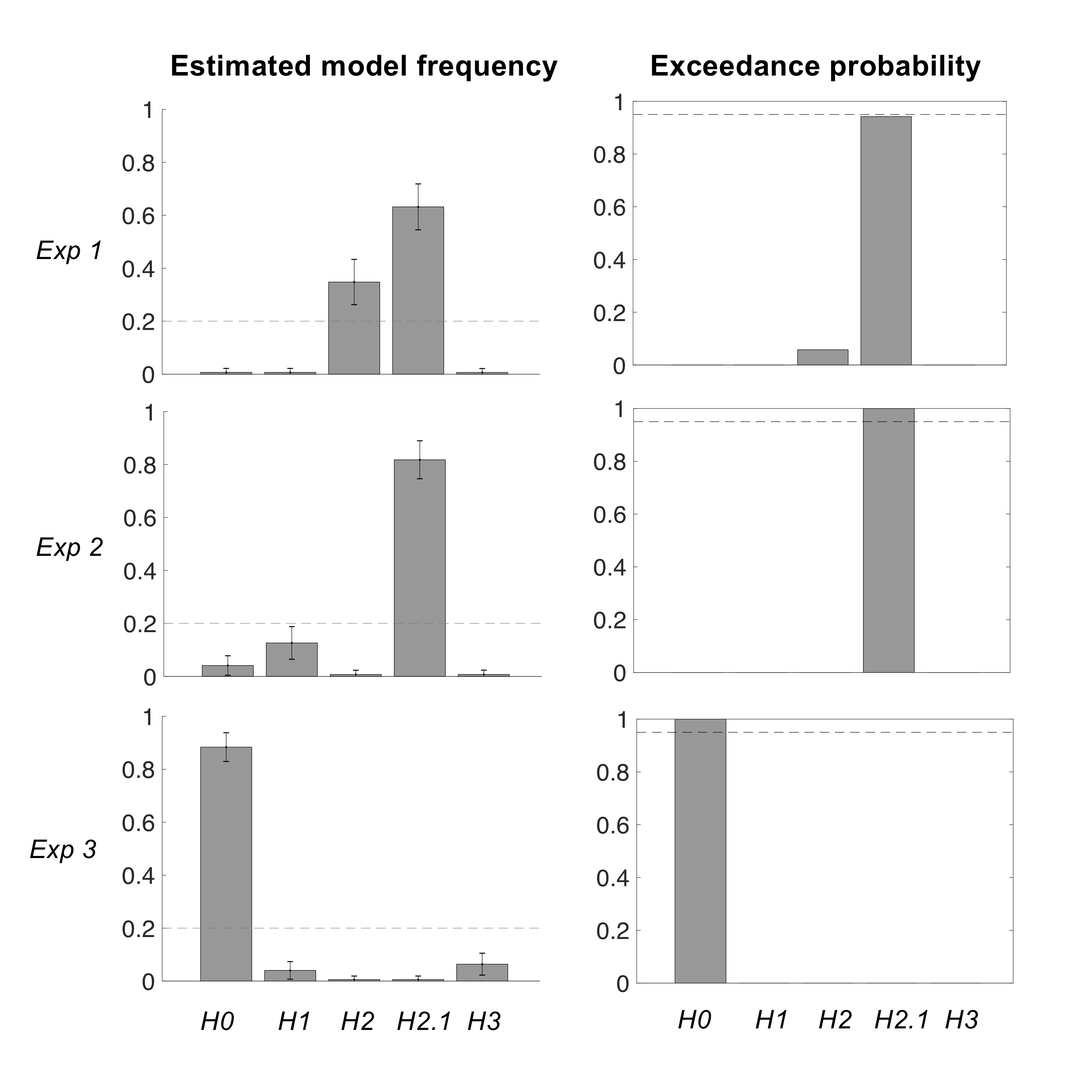

Supplement: S5 Fig — All parameters have been fitted on choice data, separately for the three experiments. Models correspond to the different hypotheses (H0 to H3). The H3 model in the plot is the pruning model, which eliminates the option losing covert pairwise comparison at each step of the sampling process. Exceedance probability is the likelihood that the considered model is more represented than the others, in the population from which participants were recruited. Dash lines represent chance level for expected frequency (0.25 because there are four models) and significance level for exceedance probability (0.95 because of the standard statistical criterion to reject random distributions). (TIF) [file pcbi.1007920.s006.tif]

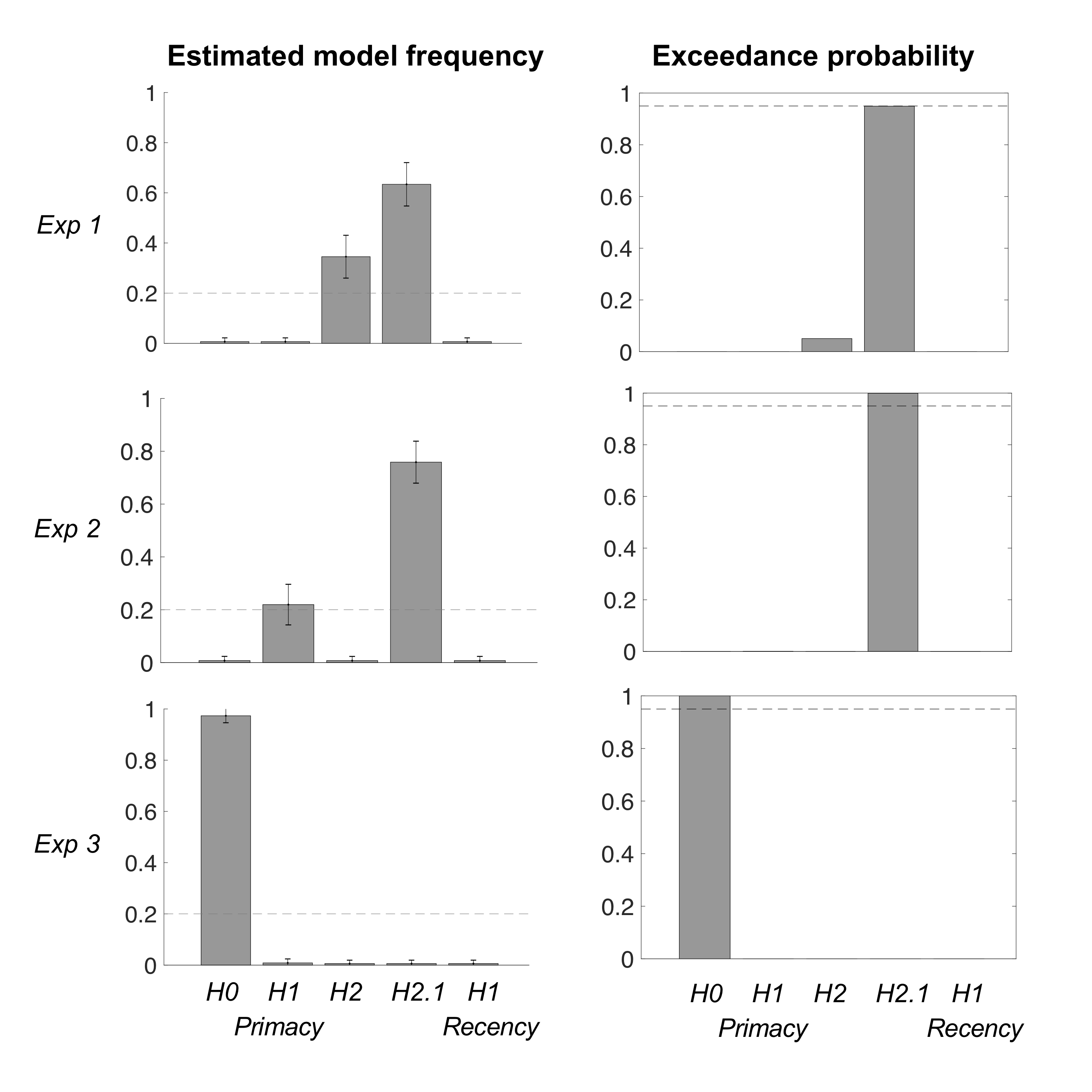

Supplement: S6 Fig — All parameters have been fitted on choice data, separately for the three experiments. Models correspond to the different hypotheses (H0 to H2.1). The H1 primacy model in the plot is the same as in the main figure, with a bias parameter bounded to be positive, thus capturing primacy effects. The H1 recency model corresponds to model H1 with a bias parameter bounded to be negative. Exceedance probability is the likelihood that the considered model is more represented than the others, in the population from which participants were recruited. Dash lines represent chance level for expected frequency (0.25 because there are four models) and significance level for exceedance probability (0.95 because of the standard statistical criterion to reject random distributions). (TIF) [file pcbi.1007920.s007.tif]
