# Supplementary material for: Order matters: How covert value updating during sequential option sampling shapes economic preference
Source: PLoS Comput Biol. 2020 Aug 11;16(8):e1007920. doi: 10.1371/journal.pcbi.1007920 (PMC7418959; doi:10.1371/journal.pcbi.1007920)
Supplement: S1 Table — (DOCX) [file pcbi.1007920.s008.docx]

# Supplementary Tables

***S1 Table. Fitted computational parameters (posterior mean*** *±* ***SEM)***

| Model | Parameter 1 (mean ± SEM) | | | Parameter 2$(mean SEM)$ | | |
| --- | --- | --- | --- | --- | --- | --- |
|  | Exp 1 | Exp 2 | Exp 3 | Exp 1 | Exp 2 | Exp 3 |
| H0 (β) | $0.086\pm0.00$71 | $0.105\pm0.0079$ | $0.093\pm0.010$ |  |  |  |
| H1 (β, λ) | $0.086\pm0.0072$ | $0.106\pm0.0079$ | $0.093\pm0.010$ | $0.40\pm0.056$ | $0.47\pm0.082$ | $0.28\pm0.043$ |
| H2 (β, δ) | $0.080\pm0.0063$ | $0.103\pm0.0089$ | $0.093\pm0.0098$ | $0.61\pm0.090$ | $0.27\pm0.098$ | $0.037\pm0.079$ |
| H2.1 (β, δ) | $0.081\pm0.0063$ | $0.103\pm0.0091$ | $0.093\pm0.0098$ | $0.59\pm0.089$ | $0.29\pm0.11$ | $0.013\pm0.087$ |
| H3 (β) | $0.077\pm0.0070$ | $0.084\pm0.0069$ | $0.074\pm0.0089$ |  |  |  |
