# Supplementary material for: Order matters: How covert value updating during sequential option sampling shapes economic preference
Source: PLoS Comput Biol. 2020 Aug 11;16(8):e1007920. doi: 10.1371/journal.pcbi.1007920 (PMC7418959; doi:10.1371/journal.pcbi.1007920)
Supplement: Attachment — Submitted filename: Hu_PlosCB_CovertPairwiseComparison_Rebuttal.pdf [file pcbi.1007920.s009.pdf]

Dear Dr Soltani and Dr Gershman,

We would like to thank all three referees for their supporting comments and for their insightful suggestions that prompted us to improve the paper. We took this opportunity to test several alternative computational models. The main conclusion remains that our covert pairwise comparison model provides the best account of choice behavior, when options are sequentially browsed through, and resampling either costly or just impossible. We have also fixed a number of presentational issues that should clarify our demonstration. As we incorporated a substantial number of new analyses, the number of figures presented as supplementary material largely exceeded what was provided in the main manuscript. To maintain balance, we have therefore integrated in the main manuscript some results that were previously illustrated in supplement information.

Please find below a point-by-point response (in blue) to the reviewers' comments (in black), detailing all the revisions made to the manuscript (highlighted in yellow). Note that following on Reviewer 2's suggestion, the order of experiments 2 and 3 has been swapped. So what we call hereafter Exp 2 and Exp 3 were previously referred to as Exp 3 and Exp 2 (respectively). These systematic changes are not highlighted in the text. Also, the changes in the figures are not apparent in the manuscript, but they are reproduced in the responses below.

We are happy to answer any further query.

Sincerely yours,
Chen Hu and Mathias Pessiglione.

Reviewer #1

Chen at al. provides evidence that choice preferences are dynamically shaped during the option sampling process. Specifically, the order in which options are sampled during decision-making tasks can influence choices. Further, they show that the computational model in which option value is iteratively updated through pairwise comparisons provided the best account of participants' choice behavior. Together, these results suggest how the sequential option sampling process can affect economic decisions. The results provide an interesting extension of the existence decision-making mechanism and should be of interest to the field. However, the manuscript in its current form has several issues that need to be addressed before it would be suitable for publication. These issues and several major points for improvement are outlined below.

→ We thank Reviewer 1 for these positive and constructive comments.

Major comments

1. It would be useful if the exposure time of each choice option (the time duration when each choice option was viewed) can also be taken into account in the analysis. Previous literature has shown that overt attention can enhance option value representation (Krajbich, et al, Nature Neuroscience, 2010; McGinty, et al, Neuron, 2016). Though the eye position signals in the current study were not recorded, it would be, nevertheless, worthwhile to investigate whether the exposure time of each choice option affects choice preferences.

→ We thank Reviewer 1 for the suggestion, we agree that exposure time could be a factor impacting the decision. The complication is that exposure time itself may be (at least partially) driven by option value (i.e., subjects may look longer at options they like better), which is the main driver of choice.

We thus started by assessing the effect of option value on exposure time. We could only do it in Exp 2, because in Exp 1 the first option was shown before any key press, making it different from the next options, and in Exp 3 all options remained visible on screen, making the exposure time difficult to estimate, as we have no recording of where subjects were looking on the screen.

The direct correlation between option value and exposure time was positive as expected, but not significant ($r = 0.031 \pm 0.0037$, $t(26) = 0.85$, $p = 0.41$). Consistently, the median split on option value suggested that subjects look longer at options with higher values, but this difference was not significant (0.89 ± 0.075 versus 0.80 ± 0.042 seconds, $t(26) = 1.46$, $p = 0.16$). This absence of effect could be related to the fact that exposure time was constrained in our experiment, as subjects had to wait for a minimum of 0.5 seconds before proceeding to the next option.

We nevertheless tested whether exposure time (ET) would impact the choice of the option with highest value (i.e., the best option) in a logistic regression that also included decision value (DV), using the following softmax function:

$$P_{best} = 1/\left(1 + exp(-(\beta_1 DV_{best-others} + \beta_2 ET_{best}))\right)$$

Regression estimates were significant for DV, as expected (β_1= 0.030 ± 0.0019, t (26) = 15.68, p < 0.001), and also for ET (β_2 = 0.34 ± 0.13, t(26) = 2.69, p = 0.012). Thus, as Reviewer 1 correctly guessed, exposure time was indeed contributing to the probability of choosing the best option.

In principle, this could have confounded our main effect, if the best option was looked longer when shown earlier in the sequence. However, this was not the case (see figure below, in which exposure to the best option is plotted as a function of its position). If we test the slopes of exposure time, as we tested the slopes of choice probability, there was no effect (b = 0.0026 ± 0.012, t(26) = 0.22, p = 0.83).

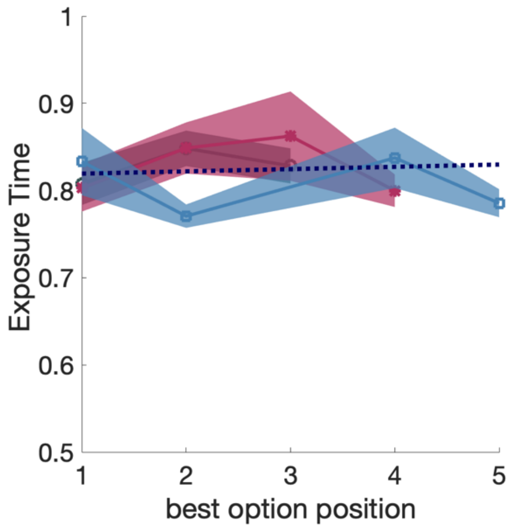

We therefore conclude that the effect of exposure time on choice probability is real but orthogonal to the main focus of our manuscript (i.e., the impact of serial position in a sequence). We nonetheless report it in the revised results section (pages 20-21), with citations of the studies mentioned by Reviewer 1, as follows:

"A possible confound for the effect of serial position is exposure time, which has been found to bias decision-making in previous studies (Krajbich, et al, Nature Neuroscience, 2010; McGinty, et al, Neuron, 2016). Here, we could only assess the impact of exposure time in Exp 2, because in Exp 1 the first option was shown before any key press, making it different from the next options, and in Exp 3 all options remained visible on screen, making exposure time difficult to estimate without tracking gaze fixation. When choice probability was regressed against a logistic

model that contained both decision value (DV) and exposure time (ET), the two regressors were found to have a significant influence (DV: b = 0.030 ± 0.0019, t (26) = 15.68, p < 0.001; ET: b = 0.34 ± 0.13, t(26) = 2.69, p = 0.012). The dissociation between exposure time and option value is not easy to operate however, because participants tend to look longer at options they like better in the first place.

In any case, we checked that the putative impact of exposure time could not contribute to the observed effect of serial position. This would occur if the best option was looked longer when presented earlier in the sequence. It was not the case: when regressing exposure time against the best option serial position, just as we did for choice probability, the slopes were not different from zero (b = 0.0026 ± 0.012, t(26) = 0.22, p = 0.83). Therefore, even if longer exposure time indeed contributed to a higher choice rate, this effect was orthogonal to our main research interest (i.e., the impact of serial position during sequential sampling)."

2. More models need to be compared to solidify the results. For example, in Exp 3, H1 shows slightly better performance than H2. To better demonstrate the main results (Figure 4). It is useful to compare the performance between H1.1 and H2.1. ***Here, H1.1 is defined the same as H1 with an additional bonus for the first option.*** Besides, it is also not clear whether other forms of primacy model can better explain the behavior data. For example, V_i (t_1)=V_i (t_0)×(1+λ/s_i).

→ We agree this is a fair point: the results of Bayesian model comparison may depend on the particular function we used for the primacy bias. In our initial analyses, we had tested two different functions:

$$V_i(t_1) = V_i(t_0) + \frac{\lambda}{s_i} \qquad (1)$$

$$V_i(t_1) = V_i(t_0) + \lambda^{s_i} \qquad (2)$$

With V_i and S_i being the value and serial position of option i, and λ a free parameter that adjusts the magnitude of the primacy bias. As the inverse function of serial position (1) outperformed the exponential function (2), we kept the former for our main model comparison.

Following on Reviewer 1's suggestion, we have implemented two alternative models for the primacy bias:

$$V_i(t_1) = V_i(t_0) \times \left(1 + \frac{\lambda}{s_i}\right) \qquad (3)$$

$$V_i(t_1) = V_i(t_0) + \lambda_1 + \frac{\lambda}{s_i} \text{ if } S_i = 1 \text{ and } V_i(t_1) = V_i(t_0) + \frac{\lambda}{s_i} \text{ if } S_i > 1 \qquad \text{(H1.1)}$$

Model 3 is just another decay function of serial position, while model H1.1 is the closest to our winning model, the first option receiving an additional bonus (λ_1). Still, our covert pairwise comparison model H2.1 outperformed both model 3 (Exp 1: Ef = 0.98, Ep > 0.99; Exp 2: Ef = 0.98, Ep > 0.99) and model H1.1 (Exp 1: Ef = 0.96, Ep > 0.99; Exp 2: Ef = 0.62,

Ep = 0.91). As model H1.1 might be penalized by having an additional parameter, we have also tried a variant with λ_1 replaced by λ (so the bonus for the first option is now 2λ), but the model comparison yielded similar results.

In conclusion, our favorite model H2.1 would be selected as the best model, irrespective of which primacy model it is compared to. We have included these additional analyses, which indeed solidify our conclusions, in the revised manuscript.

Model 3 has been included in the preliminary comparison between decay functions made to identify the best primacy model, which was then included in the main model comparison (page 22), as follows:

"In order to give the best chance to the primacy model, we first compared, after Exp 1, the different possible functions relating the primacy bias to the option serial position (i.e., H1, H1a and H1b). The results of Bayesian model selection suggested that H1 was the most plausible function (Ef = 0.76, Ep > 0.99). In the model space, we therefore included null, primacy and bonus models (H0, H1, H2, and H2.1), but not the pruning model (H3), since it predicted a trend opposite to that observed in the data. For similar reasons, we bounded the prior of the bias parameter in the primacy model (H1) to be positive, because we did not observe any trend in the data that would reflect a recency bias."

Model H1.1 has been included in a series of control analyses meant to check that model H2.1 was still winning against more subtle variants of competing models (page 25):

"Second, we compared model H2.1 to a model H1.1 where the first item gained an extra bonus $\lambda 1$, relative to all subsequent items:

$$V_i(t_1) = V_i(t_0) + \lambda_1 + \frac{\lambda}{s_i} \text{ if Si} = 1 \text{ and } V_i(t_1) = V_i(t_0) + \frac{\lambda}{s_i} \text{ if Si} > 1$$

The idea was to reduce the difference between models H1 and H2.1 to the critical impact of covert pairwise comparison. This model H1.1 would not produce a linear relationship between best-option choice rate and serial position, but simply accentuate its convexity. Expectedly, model H2.1 won the comparison with model H1.1 in both Exp 1(Ef = 0.96, Ep > 0.99) and Exp 2 (Ef = 0.62, Ep = 0.91). Because H1.1 might have been penalized for having one more parameter, we also tried a variant of H1.1 with $\lambda 1$ replaced by λ (so the bonus for the first option is now 2λ), but the model comparison yielded similar results."

Minor comments

1. To visualize the effect in Exp 3, it would be helpful to plot P(best) as a function of its absolute serial position, similar to Exp1.

→ We thank Reviewer 1 for bringing up this point. In the initial version, we adjusted plotting to the factor that was controlled (i.e., absolute position of best option in Exp 1 and relative position of best and second-best option in Exp 2 and Exp 3). However, we see the advantage of showing the same plot for all experiments, so they are easier to compare visually. We now provide these plots in the revised version of the results (see new Figure 3 below). Note that regressing P(best) against absolute and not relative position does not change the result: regression coefficient was significantly negative in Exp 2 (b = - 0.020 ± 0.0084, t(32) = - 2.32, p = 0.028) but not in Exp 3 (b = -0.0042 ± 0.0063, t(26) = -0.67, p = 0.50).

To make visual comparison easier, we have also plotted the simulated choices in the same way. The slopes of simulated choices are similar to those of observed choices, in every experiment.

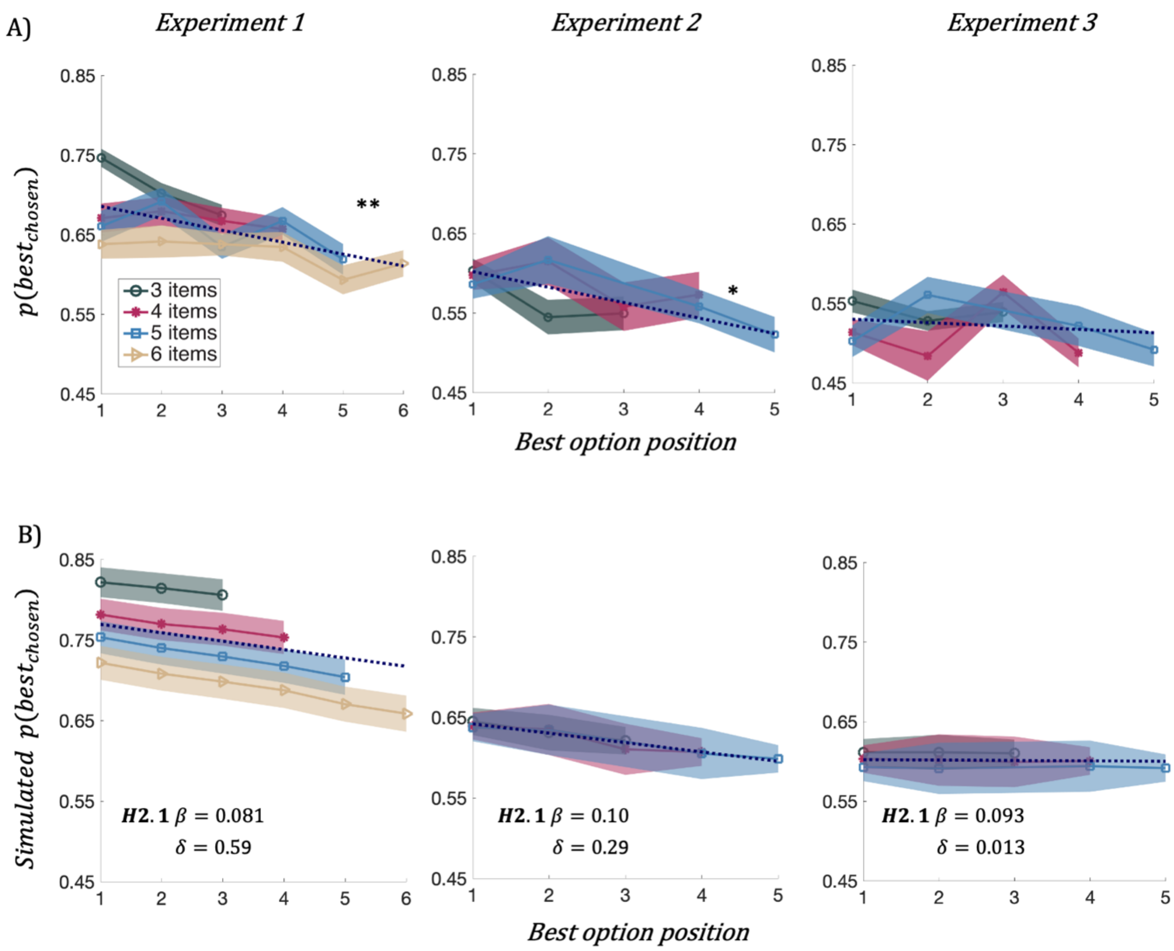

Fig. 3 – Comparison of behavioral results to model simulations.

A) The upper graphs show the observed probability of choosing the best option, as a function of its serial position, for different (color-coded) number of options. Shaded areas indicate inter-participant SEM. Dotted lines show linear regression fit across all trials (with different numbers of options). Stars denote significance of t-test comparing regression slopes to zero. * p<0.05, ** p<0.01.

B) The bottom graphs show the simulated probability of choosing the best option, as a function of its serial position. Choice behavior in each condition was simulated using the best-fitting model with the posterior means for free parameters (see values of the inverse temperature β and bonus δ indicated on the plots). Each of the plots is an average over 200 simulated datasets of 30 subjects implementing the corresponding model, for various (color-coded) number of options. Shaded areas indicate the average inter-participant SEM. across all datasets.

For the sake of consistency, we have also updated Figure S1 (see below), such that model simulations for Exp 2 and Exp 3 are plotted as a function of absolute (not relative) serial position, as was done in Exp 1 (Figure 2B).

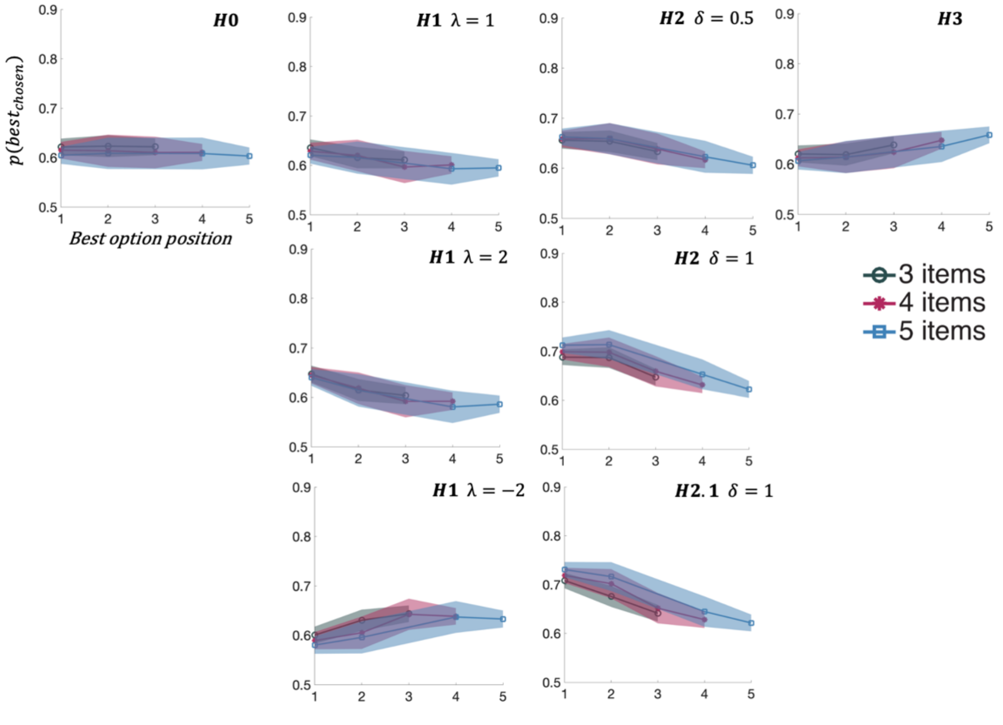

Fig. S1 - Simulations of choice data (under the settings of Exp 2 & 3).

Graphs show simulated probability of choosing the best option under the experimental setup of Exp 2 and 3, depending on the serial position of the best option in the sampling sequence (x-axis). Each of the plots is an average over 200 simulated datasets of 30 subjects implementing the corresponding model, for various (color-coded) number of options. Shaded areas indicate the average inter-participant SEM, across all datasets. Values of λ and δ are indicated on the plots. The inverse temperature parameter was fixed to β = 0.10, which corresponds to the posterior estimates of the best model (H2.1) fitted to choice data in Exp 2. The simulations show that predictions about the link between P(best) and its serial position are similar to those made under Exp 1 settings. In particular, only H1 (with a positive bias) and H2 (including H2.1) predict a decreased choice rate when the best option is presented later in the sequence.

Finally, we have kept the plots showing best-option choice rate as a function of the relative serial position (between best and second-best options) as a supplementary figure (Fig. S2 below).

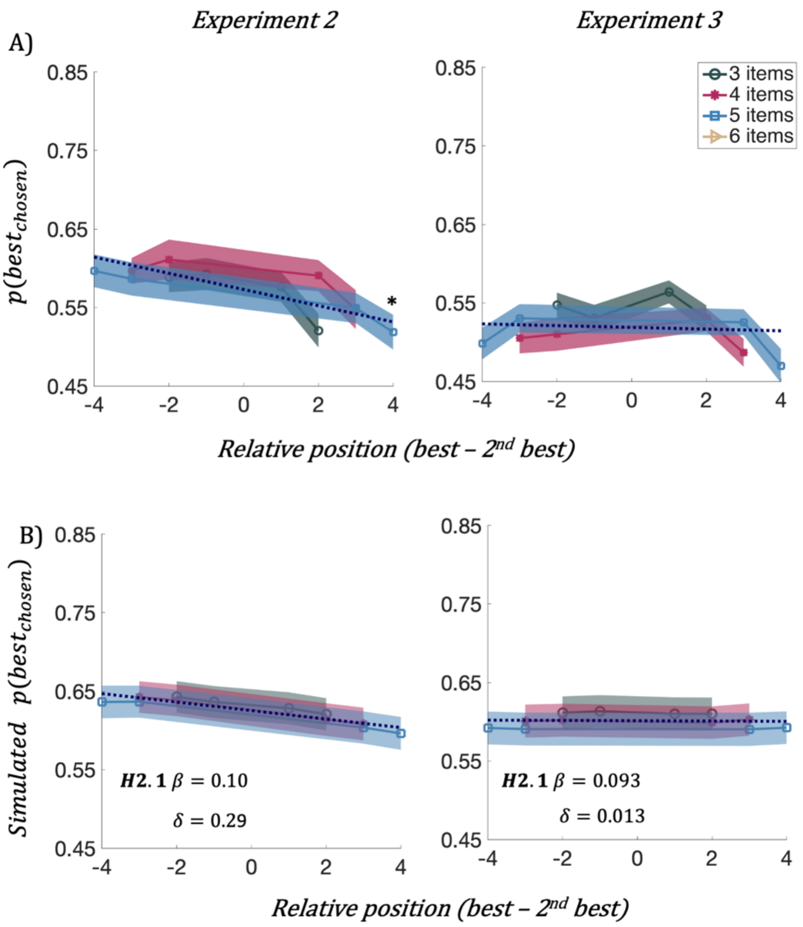

Fig. S2 – Comparison of behavioral results to model simulations in Exp 2 & 3.
A) The upper graphs show the observed probability of choosing the best option, as a function of its serial position relative to that of the second-best option. A positive relative position means that the best was presented after the second-best option. Shaded areas indicate inter-participant SEM. Dotted lines show linear regression fit across all trials (with different numbers of options). Stars denote significance of t-test comparing regression slopes to zero. * $p<0.05$, ** $p<0.01$.
B) The bottom graphs show the simulated probability of choosing the best option, as a function of its serial position. Choice behavior in each condition was simulated using the best-fitting model with the posterior means for free parameters (see values of the inverse temperature β and bonus δ indicated on the plots). Each of the plots is an average over 200 simulated datasets of 30 subjects implementing the corresponding model, for various (color-coded) number of options. Shaded areas indicate the average inter-participant SEM. across all datasets.

2. In the last part of the results, the author shows that the two-level model H2.1 provided a better account than local model H2.1 in both Exp 1 and Exp 3. In order to better illustrate the overt choice effect in all experiment condition, it would be useful to test whether the two-level H0 model also best explain Exp2.

→ We thank Reviewer 1 for this suggestion. We have included Exp 2 (now Exp 3) in the revised Figure 6 (see below), where two-level models are compared to local and global models. To maintain consistency in the figure, we fitted Exp 3 with the different variants (local, global and two-level) of model H2.1. As Reviewer 1 correctly assumed, the two-level variant best explains choices in Exp 3 (Ef = 0.98, Ep > 0.99), as it does for Exp 1 and Exp 2.

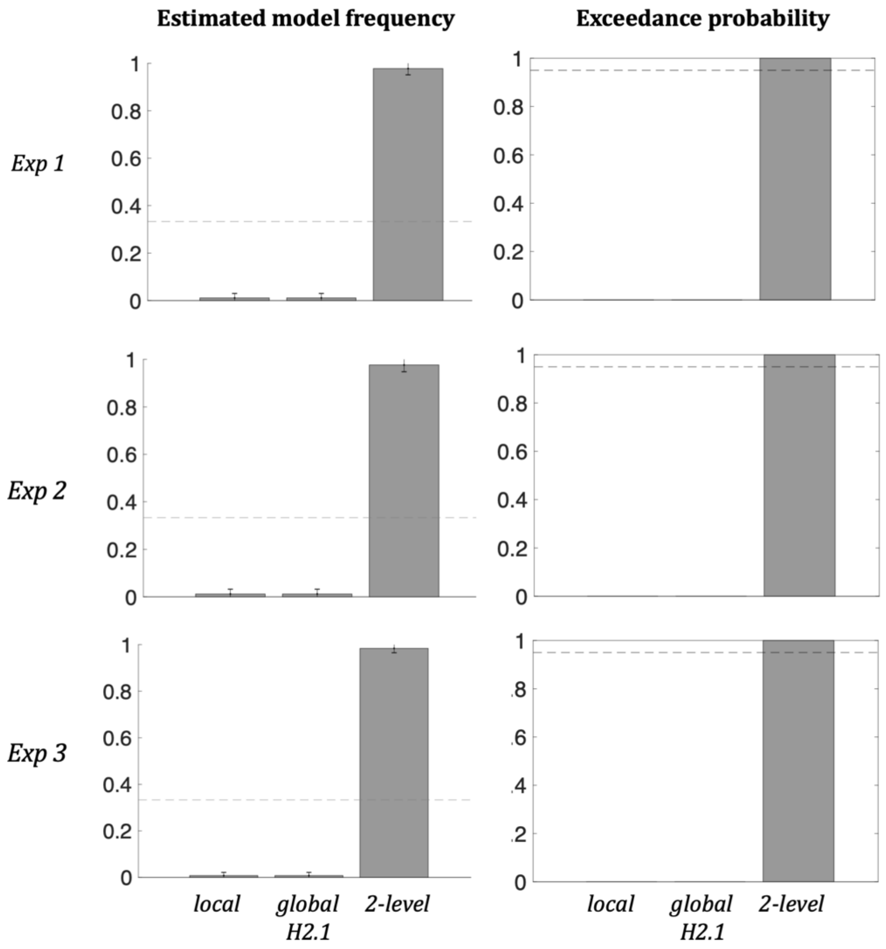

Fig. 6 – Bayesian model comparison between variants of model H2.1.
All parameters have been fitted on choice data, separately for three experiments. The model H2.1 implemented in previous comparisons is what we call here local model H2.1, which compared to two variants: the global model H2.1, in which value updates related to covert pairwise comparisons are carried over to all subsequent trials, and a two-level model H2.1, in which value updates induced by covert comparisons remain local, whereas value updates induced by overt comparisons (actual choices) are carried over to subsequent trials. Note the two-level model uses different bonus parameters for updating values after covert and overt comparisons. In the graph, gray dash lines represent chance level for expected

frequency (0.33 because there are 3 models) and significance level for exceedance probability (0.95 because it corresponds to standard statistical criterion).

However, we understand that this comparison is not exactly that requested by Reviewer 1 (between H0 and 'two-level H0'). We find the appellation 'two-level H0' confusing because, if we are not mistaken, the Reviewer wants a model with a bonus parameter δ for overt choice but not for covert choice. We prefer to call it a hybrid model, as it implements H0 for covert choice but H2.1 for overt choice. Note that, when fitting choices in Exp 3, two-level H2.1 is equivalent to this hybrid model, because the parameters used to update values following covert choices are close to zero. We nervertheless completed the analysis by comparing directly the hybrid model to a global H0 model, where neither covert choice nor overt choice had an influence. As anticipated by the Reviewer, the hybrid model won this comparison (Ef = 0.99, Ep > 0.99), providing evidence for an impact of overt choice on option value, even when covert choice has no impact.

We therefore stressed that the overt choice effects were present in all experiments (pages 28-29), as follows:

"Results confirmed that two-level H2.1 was the overall best model in all experiments (Exp 1: Ef = 0.98, Ep > 0.99; Exp 2: Ef = 0.98, Ep > 0.99; Exp 3: Ef = 0.98, Ep > 0.99). Note that in Exp 3, the fitted parameter δ for covert comparison was close to zero. Consistently, the two-level H2.1 model was outperformed by a hybrid model having just one bonus parameter for updating value following overt choice (Ef = 0.84, Ep > 0.99). This hybrid model also outperformed the global H0 model (Ef = 0.99, Ep > 0.99), confirming the bonus arising from overt choice, even when there was no bonus for covert choice. In the two other experiments (Exp 1 and Exp 2), where two-level model H2.1 was the winner, the posterior mean of the covert bonus parameter was much smaller than the overt bonus parameter (mean ± SEM: 0.48 ± 0.11/ 0.35 ± 0.13 compared to 24.73 ± 0.82 / 14. 08 ± 0.63 in Exp 1 / 2). Thus, although it provides a proof of concept for the process of covert pairwise comparison, the shift of preference induced during option sampling was less substantial than that induced by choice itself."

3. In Exp 3, what were the options that participants resampled? Did resampling increase confidence?

→ This is an interesting point but difficult to address properly given the rarity of resampling in our data. The average proportion of trials with resampling was about 12%, but this proportion varied across participants. We nevertheless tried to identify a pattern in resampling behaviour. There was no clear spatial and temporal pattern (meaning that options at a given spatial or serial position would be resampled more). However, compared to base rate (33, 25 and 20% for 3-, 4- and 5-item sequence), the best and second-best options were resampled significantly more often ($t(25) = 5.74$, $p < 0.001$ and $t(25) = 3.04$, $p = 0{,}0054$, respectively).

We also compared confidence rating between trials with and without resampling: it was significantly lower after resampling (from 77.53 ± 1.83 to 63.22 ± 2.61, $t(50) = -4.53$, $p < 0.001$). This result does not support the idea that resampling increases confidence, but it is hard to conclude on this point, because we have no access to their confidence level before they resample. If we assume it was even lower, then the data would be consistent with the idea that participants resample when their confidence is low. This interpretation would mean that subjects resample when their confidence is low, under the impression they missed or forgot some information, and that resampling does not restore the usual level of confidence, even if it helps.

We have added the requested analyses to the new results section (pages 21-22), but preferred to remain cautious about the interpretation:

"We therefore explored resampling behavior in more details, in Exp 2 where we could track it, although it remained rare (12% of trials on average). There was no clear spatial or temporal pattern: options presented at a given location on screen or at a given position in the sequence were not resampled more often than the others. However, compared to base rate (33, 25 and 20% for 3-, 4- and 5-item sequence), the best and second-best options were resampled significantly more often ($t(25) = 5.74$, $p < 0.001$ and $t(25) = 3.04$, $p = 0.0054$, respectively). We also compared confidence rating between trials with and without resampling: it was significantly lower after resampling (from 77.53 ± 1.83 to 63.22 ± 2.61 %, $t(50) = -4.53$, $p < 0.001$). This result does not support the idea that resampling increased confidence, but it is hard to conclude on this point, because we have no access to their confidence level before participants start resampling. Thus, the data remain consistent with the possibility that participants resampled when their confidence was low, under the impression that they missed or forgot some information, with this resampling behavior not restoring their usual confidence level (even if it helped). However, no strong conclusion about resampling should be drawn here, because it was only observed in a limited subset of trials and subjects."

4. The labels and the legends in Fig. 3 and Fig. S1 are mislocated or missing. Shade areas for S.E.M. are missing in Fig. 3 and Fig. S2.

→ We apologized for this incident, which happened during the automatic conversion to PDF format. We have made sure this would not occur when submitting the revised version.

5. Fig. S1 is not mentioned in the main text.

→ We thank Reviewer 1 for noticing this omission, now corrected (page 14, line 14).

Reviewer #2

In their paper "Order matters: how covert value updating during sequential option sampling shapes economic preference", Hu and colleagues investigate whether the order of presenting choice options in a multi-alternative choice task influences decisions. In three experiments, several variants of a task are tested, in which up to 6 choice options are presented sequentially. The central finding is that - contrary to standard economic theory - participants are more likely to choose the (subjectively) best option if this option occurs early in the sequence. Using computational modeling, the authors show that this effect is unlikely to be caused by a (memory-related) primacy effect. Instead, a model that assumes that options are compared in a covert pairwise manner and that the "winner" of this comparison receives a bonus to its subjective value explains the data best (at least under mnemonic constraints).

Overall, I am very enthusiastic about this paper which is well and succinctly written and addresses an important and timely research topic, which is the computational basis of seemingly irrational behavior in decisions between multiple (i.e., >2) alternatives. In many everyday life situations, such as grocery shopping, we are faced with a vast number of choice options, and it is unlikely that we can process all choice options at once. Thus, we are forced to search sequentially through the alternatives, and the experiments by Hu and colleagues create such situations in the lab. In my view, the combination of behavioral analyses and computational modeling shows in a convincing manner, that participants engage in covert pairwise comparisons and that these comparisons influence the final decision – a finding that may have substantial impact on research in various fields such as neuroscience, psychology and economics. Below are a few major and several minor suggestions for improving the manuscript.

→ We would like to thank Dr. Gluth for these constructive and positive comments.

<u>Major comments:</u>

1. As far as I understand, the "bonus" models (H2 and H2.1) assume that the option with the higher rating always wins the covert comparison and thus always receives the positive bonus. This may not be a very plausible assumption, given that we know that choices are always probabilistic (and that the model itself assumes a probabilistic choice process at the very end – see equation 1). Thus, it would be more plausible to assume that the probability that an option A wins the pairwise comparison against another option B depends on the value difference between A and B (quantified again by a Softmax function, possibly with a different inverse temperature parameter than the one used in equation 1). Obviously, this makes things a bit more complicated because the winner of a pairwise comparison cannot be specified deterministically anymore. So, one has to take all potential paths of pairwise comparisons into account (plus the probability of these paths being realized) in order to specify the predictions of the model. Nevertheless, figuring this out should not be too difficult, and I would ask the authors to consider this model in addition to their existing set of

models.

→ We thank Dr Gluth for suggesting this probabilistic approach, which we agree makes sense. The deterministic covert comparison implemented in our model H2.1 is clearly a simplification that we used to establish a proof of concept. We followed on the suggestion and calculated the probability of taking every possible path in the series of covert pairwise comparisons occurring in a given trial. This was done through a softmax function of option values, with a different inverse temperature parameter (as suggested by the Reviewer), which we call βc (keeping β for overt choice inverse temperature).

To keep the explanation as simple as possible, we only detail this calculation for a 3-item trial, where the initial values of the 3 options are noted $V_1(t_0)$ to $V_3(t_0)$. The new probabilistic model implements the following steps:

1) When the first option is shown, it receives a bonus δ (for being better than nothing), and becomes $V_1(t_1)$.

2) Then the second option is covertly compared to the first option. There are two possible winners:

- Either the first option, with probability :

$$p_{(1|1,2)} = \frac{exp(V_1(t_1) \times \beta_c)}{exp(V_1(t_1) \times \beta_c) + exp(V_2(t_0) \times \beta_c)}$$

leading to value update:

$$V_1(t_2^1) = V_1(t_1) + \delta,$$
$$V_2(t_1^1) = V_2(t_0) - \delta$$

- Or the second option, with probability:

$$p_{(2|1,2)} = \frac{exp(V_2(t_0) \times \beta_c)}{exp(V_1(t_1) \times \beta_c) + exp(V_2(t_0) \times \beta_c)}$$

leading to value update:

$$V_1(t_2^2) = V_1(t_1) - \delta,$$
$$V_2(t_1^2) = V_2(t_0) + \delta$$

3) Then the third option is covertly compared to the current best (either the first or second option), so we have now 4 possibilities for value updating. As this is

the final step in a 3-item trial, updated option values are denoted $V_1(t_{final})$, $V_2(t_{final})$ and $V_3(t_{final})$. The 4 possible paths are:

- Option 3 is covertly compared with option 1, option 1 wins, with probability:

$$p_{(1|1,3)} = \frac{exp(V_1(t_2^1) \times \beta_c)}{exp(V_1(t_2^1) \times \beta_c) \ + \ exp(V_2(t_1^1) \times \beta_c)}$$

leading to value update:

$$V_1(t_{final}^1) = V_1(t_1) + 2 \times \delta,$$
$$V_2(t_{final}^1) = V_2(t_0) - \delta,$$
$$V_3(t_{final}^1) = V_3(t_0) - \delta$$

The global probability of this first path is:

$$p_1 = p_{(1|1,2)} \times p_{(1|1,3)}$$

- Option 3 is covertly compared with option 1, option 3 wins, with probability:

$$p_{(3|1,3)} = \frac{exp(V_3(t_0) \times \beta_c)}{exp(V_1(t_2^1) \times \beta_c) \ + \ exp(V_3(t_0) \times \beta_c)}$$

leading to value update:

$$V_1(t_{final}^2) = V_1(t_1),$$
$$V_2(t_{final}^2) = V_2(t_0) - \delta,$$
$$V_3(t_{final}^2) = V_3(t_0) + \delta$$

The global probability of this second path is:

$$p_2 = p_{(1|1,2)} \times p_{(3|1,3)}$$

- Option 3 is covertly compared with option 2, option 2 wins, with probability:

$$p_{(2|2,3)} = \frac{exp(V_2(t_1^2) \ \times \beta_c)}{exp(V_2(t_1^2) \ \times \beta_c) \ + \ exp(V_3(t_0) \times \beta_c)}$$

And value updating:

$$V_1(t_{final}^3) = V_1(t_1) \ - \delta,$$
$$V_2(t_{final}^3) = V_2(t_0) + \ 2 \times \delta,$$
$$V_3(t_{final}^3) = V_3(t_0) - \delta$$

The global probability of following this path is:

$$p_3 = p_{(2|1,2)} \times p_{(2|2,3)}$$

- Option 3 is covertly compared with option 2, option 3 wins, with probability:

$$p_{(3|2,3)} = \frac{exp(V_3(t_0) \times \beta_c)}{exp(V_2(t_1^2) \times \beta_c) + exp(V_3(t_0) \times \beta_c)}$$

And value updating:

$$V_1\left(t_{final}^4\right) = V_1(t_1) - \delta,$$
$$V_2\left(t_{final}^4\right) = V_2(t_0),$$
$$V_3\left(t_{final}^4\right) = V_3(t_0) + \delta$$

The global probability of this path is:

$$p_4 = p_{(2|1,2)} \times p_{(3|2,3)}$$

At the choice stage, the probability of selecting a particular option k is calculated with the softmax function:

$$p(V_k(t_{final}^j)) = p_j \times \frac{exp(V_k(t_{final}^j) \times \beta)}{\sum_1^3 exp(V_k(t_{final}^j) \times \beta)}$$

where $V_k(t_{final}^j)$ represents the final updated value of item k following a certain path j. The overall probability of choosing the option is then obtained by summing over all possible paths:

$$p(V_k) = \sum_1^4 p\left(V_k\left(t_{final}^j\right)\right)$$

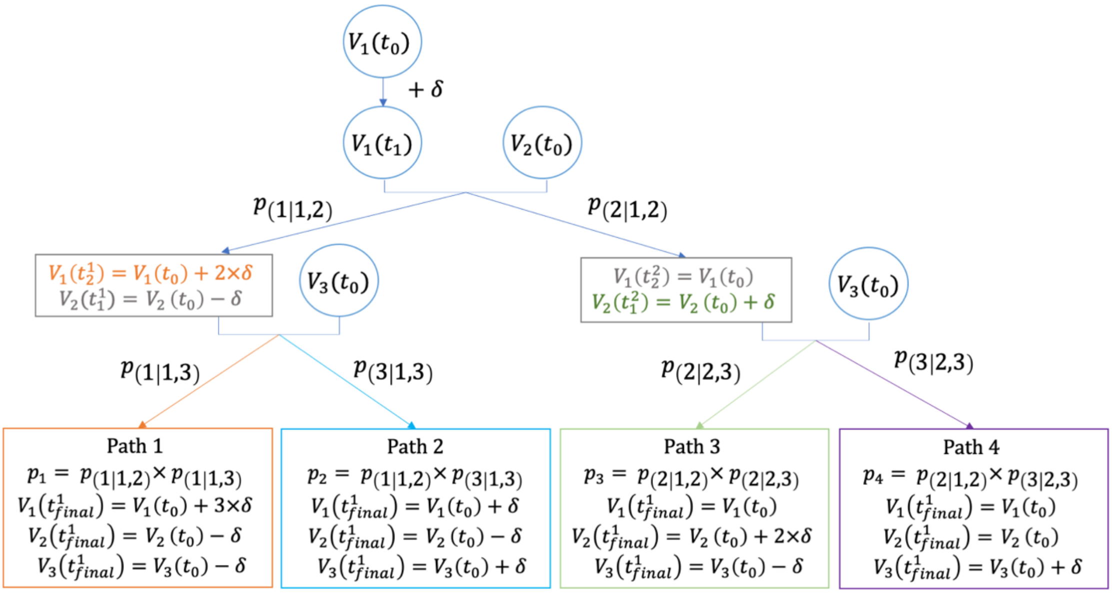

The reasoning is the same for trials with more items, which just lead to more possible combinations (precisely: 8, 16 and 32 possible paths for 4-, 5-, and 6-item trials).

When fitting this new model to choice data, we obtained different posteriors for inverse temperature parameters (Exp 1: $\beta c = 0.24 \pm 0.047$, $\beta = 0.082 \pm 0.0065$; $\delta = 0.57 \pm 0.13$; Exp 2: $\beta c = 0.22 \pm 0.049$, $\beta = 0.10 \pm 0.009$; $\delta = 0.41 \pm 0.14$). This may suggest that covert comparisons were more deterministic than overt comparisons. When comparing directly our best model H2.1 with the new probabilistic model, H2.1 remained a clear winner (Exp 1: Ef = 0.87, Ep > 0.99; Exp 2, Ef = 0.92, Ep > 0.99).

Thus, although we agree a probabilistic covert comparison is theoretically plausible, there is no evidence in our data set that would justify such degree of sophistication. Note that H2.1 and the probabilistic model yields similarly positive estimates for δ. Thus, going for probabilistic covert comparisons would not change the main conclusion that options winning / losing covert comparisons receive a positive / negative bonus is thus unchanged.

We therefore opted for keeping the winning (deterministic) model H2.1 in the main manuscript. The description of the probabilistic model H2.1 provided above has been entirely integrated in the revised manuscript (including the illustration) as supplementary information. It is also mentioned in the main result section (page 26), as follows:

“Third, we compared H2.1 to a model that we call probabilistic H2.1, because the outcome of covert pairwise comparison is probabilistically determined by a second softmax function, with a second inverse temperature parameter βc (different from the β parameter used in the softmax function that generates the probabilities of overt choices). This probabilistic version of H2.1 would be more coherent, as there is no reason to assume that covert choice is deterministic (meaning

that the best option is always winning the comparison), while overt choice is known to be probabilistic. This makes the computations much heavier, as there are many paths in the tree of possible combinations (8, 16 and 32 for 4-, 5- and 6-item trials, respectively). For each possible path, values are updated in the same manner as in the deterministic model H2.1, and passed through the same final softmax function to generate choice probabilities. Then, for every option, the overall selection probability is obtained by summing the product of path and choice probabilities over all possible paths. These computations are detailed and illustrated in supplementary methods.

The deterministic version of H2.1 won the comparison with the new probabilistic one, in both Exp 1 (Ef = 0.87, Ep > 0.99) and Exp 2 (Ef = 0.92, Ep > 0.99). The fitted inverse temperature parameters suggest that indeed, covert choices were more deterministic than overt choices (Exp 1: $\beta c = 0.24 \pm 0.047$, $\beta = 0.082 \pm 0.0065$; Exp 2: $\beta c = 0.22 \pm 0.049$, $\beta = 0.10 \pm 0.009$). However, the bonus parameters were in the same range as with the deterministic model H2.1, and significantly different from zero in both experiments (Exp 1: $\delta = 0.57 \pm 0.13$; Exp 2: $\delta = 0.41 \pm 0.14$). Thus, even if using a softmax function for covert choice is theoretically more grounded, our choice dataset was not sensitive enough to benefit from this additional parameter. In any case, the impact of covert pairwise comparisons (the bonus added to the winning option and subtracted from the losing option) was significant in both the deterministic and probabilistic version of model H2.1."

2. The authors basically propose the existence of covert decision processes on the basis of behavioral data (and computational modeling). For the Discussion, I think it would make a lot of sense to ***speculate about potential physiological or neuroscientific approaches to "directly" reveal these covert decision processes.*** Although this is clearly self-serving, I would like to point the authors to our work on uncovering the covert "decision not to decide" (Gluth et al., 2013, PLOS Comput Biol). This work together with related literature (e.g., O'Connell et al., 2012, Nat Neurosci) could form the basis for such a discussion point.

→ We thank Dr Gluth for this suggestion: using neuroimaging techniques to look for more direct evidence is indeed one of the main follow-ups for our behavioral study. We have inserted a paragraph in the discussion (pages 32-33, reproduced below) that points to this line of research. We cite the suggested paper as a proof of concept, even if the neural signature of covert decisions would probably be different in our context, since there was no choice to stop or go on with sampling information.

"Although our covert pairwise comparison model was supported here by both model-free and model-based analyses, the evidence remains indirect. More direct evidence could be searched using neuroimaging techniques to track covert choices. Previous studies have shown that covert decisions to postpone a choice (during sequential sampling of information), which were inferred from computational analyses, could be uncovered from EEG signals (Gluth et al., 2013). Thus, it might be possible to decode neural representations of the current best option, such as its spatial location, either in a perceptual or in a motor format. Also, commitment to a choice (versus

postponement), in the course of sequential sampling, has been related to decision-value signals recorded with fMRI, in brain regions such as the ventromedial prefrontal cortex (Tsetsos et al., 2014). Thus, it might be possible to assess the existence of value updates, following covert comparisons, by monitoring the activity of these brain regions. In this endeavor, our model might provide the computational probes needed to identify neural mechanisms operating covert choices."

3. The order of experiments 1 to 3 appear to reflect the temporal order in which the experiments were conducted. Although this information is relevant (and should be mentioned at some point), I would still argue that it might be preferable to report experiment 2, which is kind of a control condition, either at the beginning or at the end. This would make the manuscript somewhat more accessible in my view.

→ We thank Dr Gluth for this suggestion, which indeed makes our demonstration easier to follow. We have changed the order of the experiments, as requested, and modified figures accordingly (see below for the main figures). To maintain consistency, Exp 2 and Exp 3 have been swapped everywhere in the main texts.

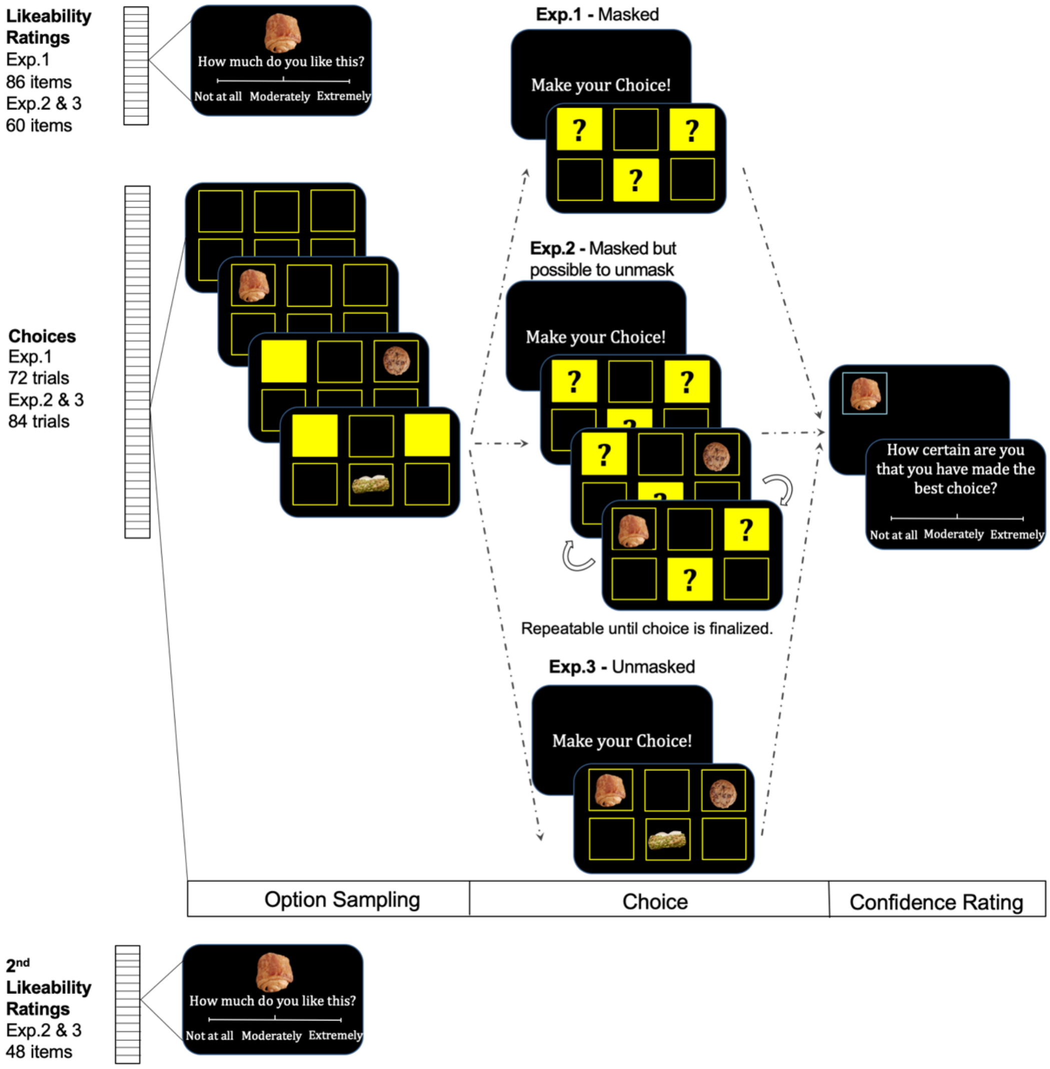

Fig. 1 - Behavioral tasks.

Each task session used one category of items (food, music, film or magazine) and was divided into two stages. In the first stage, participants had to rate the likeability of items presented one by one on the screen, by placing a cursor on a visual analog scale. In the second stage, participants had to browse through a set of 3 to 6 options, by pressing a bar on the keyboard, then click on their favorite option at the end of the sequence, and last, rate their confidence in their choice by placing a cursor on another visual scale. An additional third stage was included in Exp 2 and 3, with a second likeability rating task identical to the first one (presenting the same items). Beyond details regarding the number of items and categories, key differences between experiments regarded the choice phase: after being sampled, options remained masked (with no possibility of unmasking) in Exp 1, remained unmasked (with a time consuming possibility of resampling) in Exp 2, and were simply unmasked all together in Exp 3. Option sampling was self-paced, participants proceeding to the next option by pressing the space bar. At each step, a new option was revealed while the previous one was masked again. The location of the options appearing on screen was randomized but their identity was prearranged on the basis of likeability ratings (see methods). Choice was prompted by displaying question marks on masks (Exp 1 and 2) or by unmasking all options together (Exp 3). A feedback showing the chosen option alone was provided before the confidence rating.

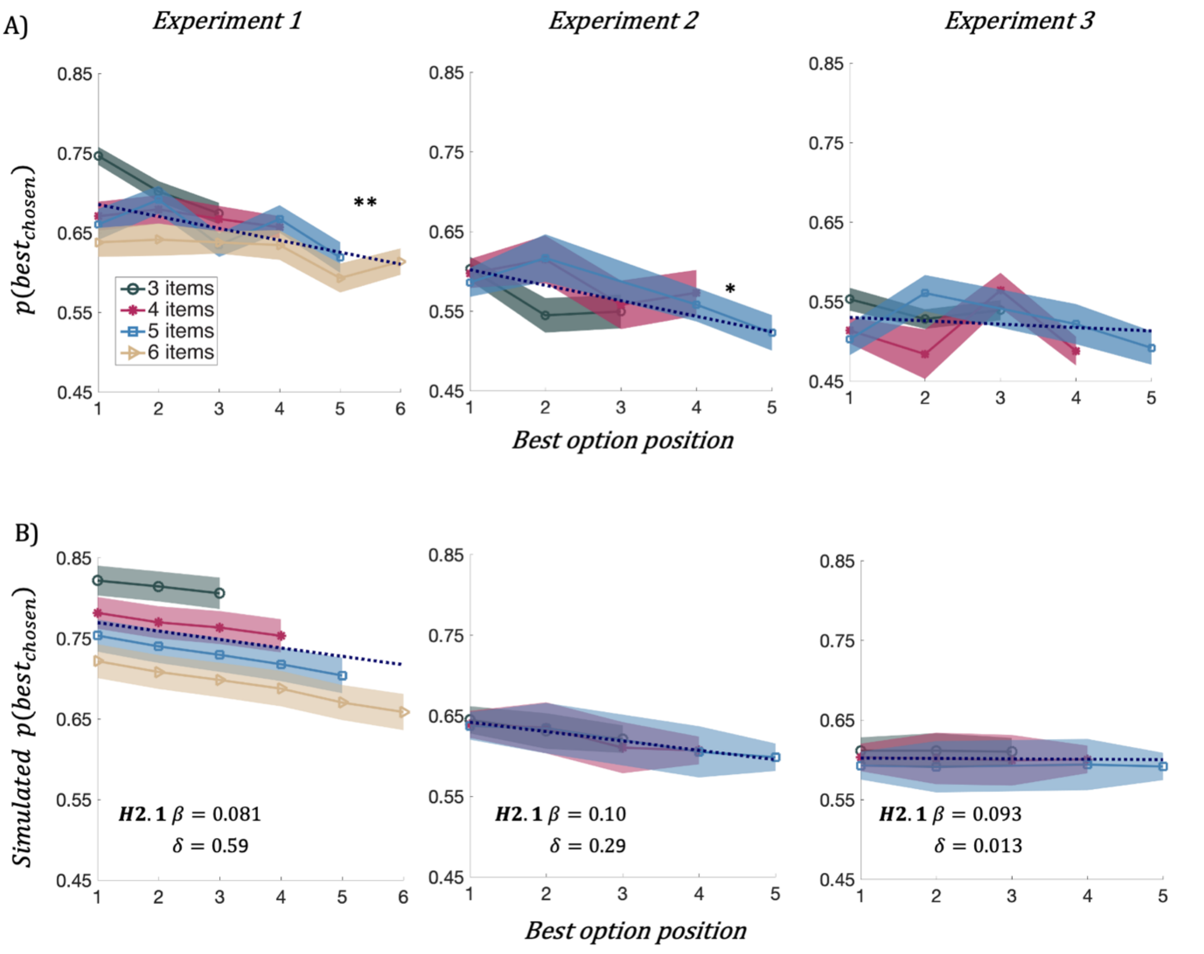

Fig. 3 – Comparison of behavioral results to model simulations.

A) The upper graphs show the observed probability of choosing the best option, as a function of its serial position, for different (color-coded) number of options. Shaded areas indicate inter-participant SEM. Dotted lines show linear regression fit across all trials (with different numbers of options). Stars denote significance of t-test comparing regression slopes to zero. * $p<0.05$, ** $p<0.01$.

B) The bottom graphs show the simulated probability of choosing the best option, as a function of its serial position. Choice behavior in each condition was simulated using the best-fitting model with the posterior means for free parameters (see values of the inverse temperature β and bonus δ indicated on the plots). Each of the plots is an average over 200 simulated datasets of 30 subjects implementing the corresponding model, for various (color-coded) number of options. Shaded areas indicate the average inter-participant SEM. across all datasets.

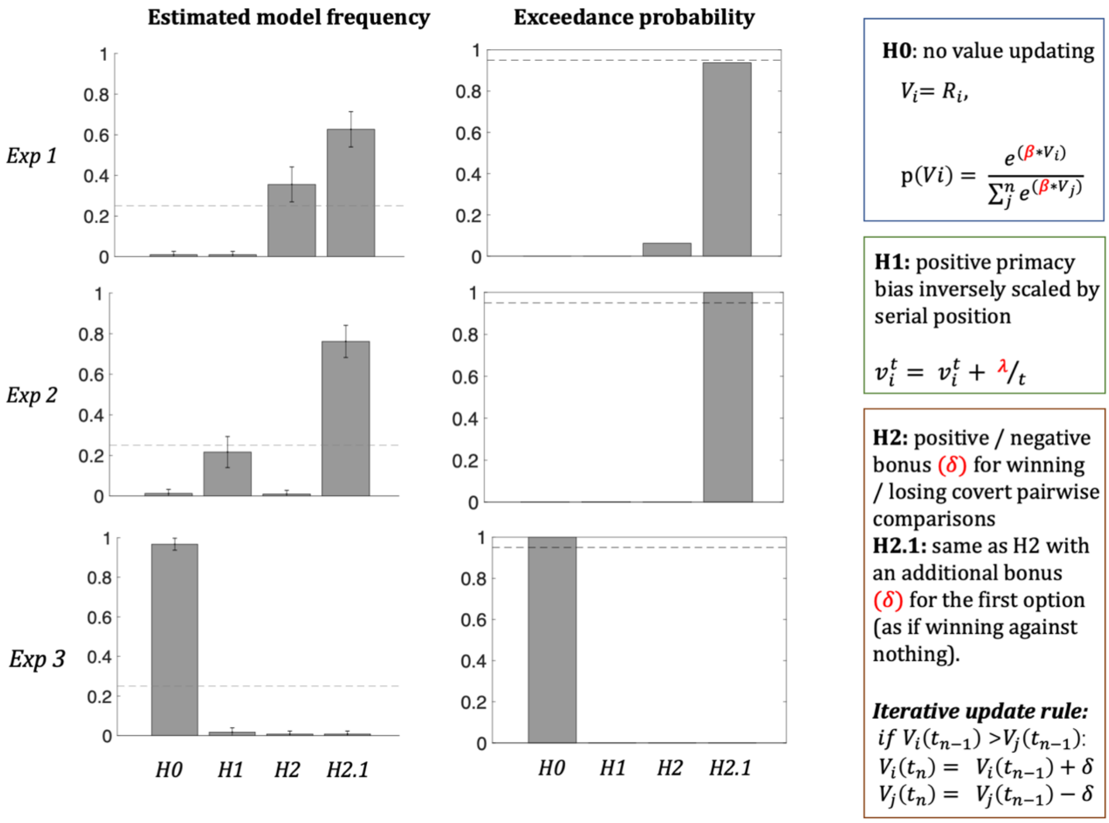

Fig. 4 - Bayesian model comparison results.

All parameters have been fitted on choice data, separately for the three experiments. Models correspond to the different hypotheses (H0 to H2.1). The bias parameter λ in model H1 was bounded to be positive, in order to capture primacy effects. The bonus parameter δ in model H2 and H2.1 was not bounded, such that a positive posterior provides evidence for the existence of covert pairwise comparison. The recency bias model (H1 with negative parameter) and the pruning model (H3) were not included because they predicted a qualitatively opposite trend (increased probability of selecting the best option when presented later in the sequence), compared to what was observed in choice data. Exceedance probability is the likelihood that the considered model

is more represented than the others, in the population from which participants were recruited. Dash lines represent chance level for expected frequency (0.25 because there are four models) and significance level for exceedance probability (0.95 because of the standard statistical criterion to reject random distributions).

We nonetheless mention the chronological order in the methods (page 8), as follows:

"The tasks are not numbered in chronological order: Exp 3 (the control experiment) was in practice conducted between Exp 1 and Exp 2 (the test experiments). Instead, for the sake of readability, they are ordered following the difficulty of resampling the options, which was made impossible in Exp 1, costly in Exp 2 and free in Exp 3 (Fig. 1)."

Minor comments:

4. Author summary (p. 3): The very first sentence needs to be rephrased, because standard economic theory does NOT assume a two-step process of valuation and decision making. This is standard NEUROeconomic theory (e.g., the chapter by Glimcher in the 1st edition of the Neuroeconomics book). Traditional microeconomics does not say anything about how (or in how many steps) decisions emerge.

→ We agree with this view and have rephrased the summary as follows:

"According to standard views in neuroeconomics, choice is a two-step process, with first the valuation of alternative options and then the comparison of subjective value estimates. Our working hypothesis is, on the contrary, that the comparison process begins during the sequential sampling of alternative options."

5. Introduction (p. 4): "…for which economic choice is construed as the selection of the option maximizing expected value". The term "value" needs to be replaced by "utility". Blaise Pascal assumed that people maximize expected value, but since Daniel Bernoulli, this has been dismissed and replaced by expected utility. In general, I don't like that the term "value" is used so often throughout the manuscript ("utility" or "subjective value" are preferable).

→ We agree that economic decision theory employs the term 'utility', but most neuroscientists use the term 'value', as in the seminal review by Rangel and colleagues (Nature Neuroscience reviews 2008), which has become a major reference for the field of neuroeconomics. We have therefore replaced value by 'utility' when we refer to economics, and explained the switch to subjective value adopted in neuroscience. We have later omitted the 'subjective' qualification when it was too cumbersome (notably in the description of computational models).

In the revised introduction, we modified the first paragraph (page 4) where we use 'utility':

"In everyday modern life, people often make choices between multiple options they can browse through, for instance when shopping for groceries, on the internet or at the supermarket. Even when options are readily available, located next to each other, they cannot be attended all at once. Sampling options is therefore a sequential process that unfolds across time, breaking equity between options by assigning them a serial position. This sequential allocation of attention is typically neglected in standard decision theory (Kahneman & Tversky, 1979; Samuelson, 1938; von Neumann & Morgenstern, 1944), for which economic choice is construed as the selection of the option maximizing expected utility. Thus, according to standard decision theory, the way information about alternative options is collected, in particular their position in the sampling sequence, should not have an impact on the eventual choice. Our working hypothesis is, on the contrary, that option sampling is not just passive information gathering, but an active process that covertly updates the utility function on which the eventual choice is based."

And then the last paragraph (pages 5-6) where we switch to 'subjective value':

"Instead of utility or preference, we employ here the term value to designate the subjective estimate of how good an outcome would be for the decision-maker, as is common in neuroeconomics (Kable & Glimcher, 2009; Rangel, Camerer, & Montague, 2008). If our hypothesis is correct, the covert pairwise comparison implemented at every step of option sampling should therefore enhance the subjective value assigned to the better option and decrease the subjective value attached to the worse option. The prediction is that the eventual (overt) choice distribution should reflect these (covert) subjective value updates. In addition, confidence in the choice, defined as the subjective belief that the (overtly) chosen option was indeed the best, should also reflect (covert) subjective value updates. This second prediction stems from previous reports that confidence increases with the distance in subjective value between chosen and unchosen options (De Martino, Fleming, Garrett, & Dolan, 2013), as it does with the distance in stimulus strength for perceptual decisions (Fleming & Lau, 2014). Crucially, our model implies that the sequence in which options are sampled will have an impact on the final choice: if the best option is encountered earlier in the series, it will win more covert comparisons, which should further boost its subjective value and hence the likelihood that this option will be selected in the end, as well as the confidence that this option was indeed the best."

6. Methods (p. 7): The number of participants (about 30 per experiment) appear to be sufficient for the purpose of the current study, but it should be noted how these numbers were determined (e.g., by a formal power analysis or by following previous studies).

→ We could not run a formal power analysis because we did know in advance the effect size. In fact, we did not know whether the effect existed, as the purpose of the experiment was precisely to test it. We just estimated that for a reasonable effect size on the bonus

parameter (similar to that obtained in previous studies), with significance at 0.05 and power at 0.9, we needed around 30 participants. This was based on a one-sample t-test (against the null $\delta = 0$, with a standard deviation around 0.5 and an effect expected around 0.3). The calculation a posteriori, given the observed effect size and standard deviation, indicates that 20 participants would have been sufficient. This is now mentioned in the methods section (page 7):

"The sample size was based on educated guess rather than formal power calculation, as we could not know in advance whether the effect of serial position would present or not. A posteriori, given the observed size of the bonus parameter and its standard deviation across participants, a formal estimation indicated that a group of n=19 participants would have been sufficient to test the effect with significance at 0.05 and power at 0.9."

7. Methods (p. 8): "Should two items have the same rating, we would compare the corresponding response time and assign a higher ranking to the one with a quicker response time". I think this "rule of thumb" only makes sense for high-value items, but the opposite should be true for low-value items. For example, let's say someone gives two options, A and B, a rating of "not at all" – but much quicker for A than for B. I would infer from this that the person is very sure that s/he doesn't like A at all but is less sure in case of B. So, my prediction for a decision between A and B would be that s/he would choose B.

→ We totally agree with this line of reasoning. However, we do not think it would affect the results in any way because:

- Rankings were only used for dealing items over trials, never for data analysis (where ratings were used instead)
- Rankings were only extracted for the best and second-best options, which by definition received a high rating, such that a shorter RT denotes a higher value. The other (less valuable) options were randomly drawn, so the reverse logic (shorter RT means lower value) never applied.

We have mentioned this point in the methods (page 9), as it would indeed be a caveat, had we used RT to rank disliked items:

"Note that rankings were only used to control the position of the best item in the sequence, never for data analysis or computational modeling, which instead were based on likeability ratings (i.e., cardinal and not ordinal values)."

8. Methods (p. 12): w.r.t. equation 1, V_j is specified as "values of the o other options". This is incorrect, because o must also include option i. Please clarify.

→ We thank the Reviewer for spotting this error. Indeed, the denominator includes all option values V_j (including V_i). This is how the function had been written in our code. We have rephrased the description in the methods (page 12), as follows:

"In every trial, selection probabilities under H0 are therefore:

$$p(V_i) = \frac{e^{\beta * V_i}}{\sum_j^o e^{\beta * V_j}} \qquad (1)$$

Where p(Vi) is the probability of choosing item i given its value Vi, and the value Vj of all the options o displayed in the trial (including i). In model H0, all option values correspond to initial likeability ratings."

9. Methods (p. 15): It would be important to know whether the free parameters were restricted to any specific ranges (esp. for the bonus parameter delta).

→ We agree this is an important information. Regarding the bonus parameter (δ), it was not bounded because we aimed to test whether the posterior was significantly positive. To avoid any bias, the prior was following a normal distribution N(0,1). Regarding the bias (λ), it was bounded to be positive when modelling a primacy bias, and negative when modelling a recency bias. This information was provided in the results section, when relevant

page 22:

"In the model space, we therefore included null, primacy and bonus models (H0, H1, H2, and H2.1), but not the pruning model (H3), since it predicted a trend opposite to that observed in the data. For similar reasons, we bounded the prior of the bias parameter in the primacy model (H1) to be positive, because we did not observe any trend in the data that would reflect a recency bias."

Page 25:

"When fitting model H2.1, the parameter δ was not constrained to be positive, so its sign can be interpreted as evidence for a bonus added to the winning option (and subtracted from the losing option)."

Page 23:

"As control analyses, we included the pruning model and the recency model (same as primacy model but with a bias parameter bounded to be negative) in the model space. "

We have now repeated the information in the legends of Fig. 4, to be sure the reader would not miss it:

"All parameters have been fitted on choice data, separately for the three experiments. Models correspond to the different hypotheses (H0 to H2.1). The bias parameter λ in model H1 was

bounded to be positive, in order to capture primacy effects. The bonus parameter δ in model H2 and H2.1 was not bounded, such that a positive posterior provides evidence for the existence of covert pairwise comparison. The recency bias model (H1 with negative parameter) and the pruning model (H3) were not included because they predicted a qualitatively opposite trend (increased probability of selecting the best option when presented later in the sequence), compared to what was observed in choice data."

10. Results (p. 18): "This suggests that using model H2.1 is adaptive in the sense that it makes decision-making closer to optimal policy on average". I think this needs to be rephrased, because theoretically it is just wrong to make this claim (model H2.1 is less optimal than H0). I suggest to avoid the term "closer to optimal" and simply say that the pairwise covert comparisons are adaptive as they help participants to deal with the cognitive demands of the task.

→ We agree that the phrasing was unfortunate. However, we are unclear why Dr Gluth suggests that "pairwise covert comparisons help participants to deal with the cognitive demand of the task". Defining optimality here is not as straightforward as the Reviewer seems to think. This is because of the softmax function, which produces some kind of matching behavior. Imagine two bandit machines that deliver 100€ with probability 0.8 and 0.2. The optimal policy would be to always choose the high-probability bandit. Thus, any choice model H0 using a softmax function with a finite inverse temperature would be suboptimal. It can therefore be adaptive to implement a mechanism that spread preferences (e.g., to expected value of 90/10 instead of 80/20). Spreading preference has the same effect as increasing the inverse temperature (i.e., it generate more exploitative choices). So, in a stable environment, a mechanism that spread preferences, like that assumed in model H2.1, would indeed been adaptive, compared to H0. Now, this would reduce exploration, hence be detrimental in non-stable environments. Thus, optimality really depends on the nature of the uncertainty in value estimation (i.e., on how much exploration is useful). To stay on safe grounds, we simply kept the idea that the covert pairwise comparison process favored the exploitation of the option with best expected value estimate. We have rephrased the incriminated paragraph to better convey this idea (page 20):

"We cannot formally compare with Exp 1, since there were several changes in the design. However, Exp 2 and Exp 3 are comparable, as they only differ in the possibility of (costly) resampling. Optimal choice (selecting the best option) was significantly more frequent ($t(58) = 3.88$, $p < 0.001$), and erroneous choice (selecting an option other than the best or the second-best) significantly less frequent ($t(58) = -2.59$, $p = 0.012$) in Exp 2 relative to Exp 3. This suggests that the putative covert pairwise comparison process implemented in Exp 2 favored the maximization of expected value, i.e. enabled a more systematic exploitation of the best option when it was encountered earlier in the sequence of option sampling. Note that resampling *per se* did not improve the best option choice rate, which was even lower in trials where participants did resample

(at least one option) and in those where they did not (t(50) = 5.20, $p < 0.001$. Yet it is difficult to conclude about the impact of resampling on choice as we do not know what the intentions of participants were before resampling."

Reviewer #3

In this paper Hu and colleagues suggest that in contrast to the traditional view in economic decision theory, during sequential option sampling people compare every new alternative to the current best. To capture this idea, authors collected data from humans performing three variants of a novel multi-alternative decision task. Authors showed that in the cases were options were provided sequentially but masked prior to presentation of a new option, subjects' choice behavior was best captured by a model in which every new alternative is compared with the current best. On the other hand, in the case where options were all unmasked at the time of choice, subjects' choice behavior did not show any effect of the sequence order.

This is a well-designed experiment with interesting results which is of importance to our understanding of sequential decision making. However, I have number of major concerns that should be addressed to strengthen the conclusions that are drawn in the paper. Please find my concerns below:

→ We would like to thank Reviewer 3 for these constructive and positive comments.

<u>Major issues:</u>

The observed behaviors can be also put into the context of attentional weighting of outcomes under risk, which has been the focus of many studies in different contexts ([1-4]). How are H2(.1) & H3 models different than these suggested models? A detailed (model-free and model-based) comparison of these models is necessary to demonstrate the novelty of authors' work.

References:

[1]. Birnbaum, M. H., & Navarrete, J. B. (1998). Testing descriptive utility theories: Violations of stochastic dominance and cumulative independence. Journal of Risk and Uncertainty, 17(1), 49-79.
[2]. Bordalo, P., Gennaioli, N., & Shleifer, A. (2012). Salience theory of choice under risk. The Quarterly journal of economics, 127(3), 1243-1285.
[3]. Farashahi, S., Azab, H., Hayden, B., & Soltani, A. (2018). On the flexibility of basic risk attitudes in monkeys. Journal of Neuroscience, 38(18), 4383-4398.
[4]. Spitmaan, M., Chu, E., & Soltani, A. (2019). Salience-driven value construction for adaptive choice under risk. Journal of Neuroscience, 39(26), 5195-5209.

→ We have been puzzled by this comment, as it is hard to see how the models developed to account for choice under risk could apply to choice between food items, where there is no events like gamble and outcome, no attributes like magnitude and probability, and no domain like gains and losses. Trying our best to make sense of the suggestion, we guessed that the Reviewer wanted us to test the role of salience-driven attention.

The idea would be that the best option is more salient than the others, and capture more attention. We have already discarded the possibility that participants spend more time examining the best option, or pay more attention to options shown early in the sequence (see our response to Reviewer 1 regarding the impact of exposure time). It remains possible that participants over-weighted the best option because it was more salient. However, this would not explain the link between best-option choice rate and its serial position, which is our main model-free result.

We also implemented a model-based refutation of this attentional hypothesis. We fitted a new model where the value of the best option within a trial was amplified due to its higher attentional salience, relative to the other options. As a consequence, the best option value was updated as follows: $V_{best} = V_{best}.(1+\gamma)$, where γ is a parameter capturing increased attentional weighting.

This model is bound to fail in explaining the impact of sequential order, as it can only account for why the best option would be chosen more frequently than what its value predicts, irrespective of its position in the sequence. Unsurprisingly, our model H2.1 won the comparison with this attentional salience model, in both Exp 1 (Ef = 0.58, Ep = 0.81) and Exp 3 (Ef = 0.98, Ep > 0.99).

We have not included this model in our description of competing computational models, because it does not address the issue of sequential presentation, which is the main focus of our paper. However, we mention it in the revised results section, with citation of suggested references, as further evidence that order matters (page 25):

"First, we compared model H2.1 to a model in which the best option simply captures attention, receiving a bonus related to its saliency, as was proposed for salient attributes in choices between lotteries (Krajbich et al., 2010; McGinty et al., 2016). This saliency model was equivalent to H0 except for the bonus γ attributed to the best option:

$$V_{best} = V_{best}.(1+\gamma)$$

This new model tests the possibility that the bonus may be independent from the serial position of the best option, and therefore cannot explain the link between best-option choice rate and serial position observed in our data. Expectedly, model H2.1 won the comparison with the saliency model in both Exp 1 (Ef = 0.58, Ep = 0.81) and Exp 2 (Ef = 0.98, Ep > 0.99). This confirms that the order of sequential sampling matters for the probability of choosing the best option."

2. It is puzzling to me why subjects' continuous likability ratings are transformed to ranks at the beginning of the experiment. I understand that ranking makes combining data from all the subjects easier. However, distances between rating are informative and affected when moving from continuous likability ratings to discrete ranks. Authors should provide evidence that transformation of non-uniform likability ratings does not affect their observed results to a large extent.

→ There is a misunderstanding here. Rankings were only used for dealing items over trials, never for data analysis or computational modeling. Ranking corresponds to the

notion of best and second-best options, which in our model is a key factor that frame the covert comparisons made by participants (every new option is compared between new and current best options). We fully agree that the distance between ratings is an important information for predicting choice probability. This is why choices were predicted through a softmax function, which translates a distance between options (along a value dimension) into a choice probability. Note that the effect of covert comparisons in our model was precisely to increase the distance between winning and losing options, relative to the initial analyses.

In the revised version, we have made clear that all model-based analyses used the ratings of the items and not their rankings (see page 9):

"Note that rankings were only used to control the position of the best item in the sequence, never for data analysis or computational modeling, which instead were based on likeability ratings (i.e., cardinal and not ordinal values)."

3. Is there a change in the slope of probability of choosing the best option as a function of position for different number of items (Figure 3A Experiment 1)? Please comment on this.

→ We are unsure what theoretical question this test would answer, but we nonetheless performed it. The Reviewer is correct: in Exp 1 the slope decreased indeed with the number of trials in the sequence ($b = 0.0080 \pm 0.0035$, $t(28) = 2.30$, $p = 0.029$). However, this pattern was not significant in Exp 2 ($b = 0.00045 \pm 0.0071$, $t(32) = 0.064$, $p = 0.95$) or in Exp 3 ($b = 0.0042 \pm 0.0062$, $t(26) = 0.68$, $p = 0.50$). Given the absence of replication, we therefore prefer not to base strong conclusions on this observation, which could relate to any peculiarity of Exp 1, or simply be a false positive.

4. Authors provide behavioral evidence supporting and against three models in their model-free analysis and later use model-based analysis to confirm those predictions. However, a few models were tested on the choice data without any behavioral evidence. ***This begs the question whether these different models are well identifiable in the experiments.*** To test this, authors should simulate each model with a range of parameters, and fit the simulated behavior with each model, then show that each model fits better data simulated by the same model.

→ We thank the Reviewer for this important suggestion. We totally agree that model recovery analysis is good practice. This analysis is a bit tricky when models are nested, because recovery depends on the parameter used. For instance, there would be no point in trying to recover model H1 (primacy model) if the bias parameter (λ) was close to zero, because it would then be equivalent to the null model. The same reasoning would apply

to the difference between H1 and H2.1, which vanishes when the bonus parameter (δ) approaches zero. Following common practice, we reasoned that what we want to know is whether the Bayesian model comparison can distinguish between models using parameters pertaining to the range observed in our pool of participants. We therefore simulated choices in 50 groups of 30 subjects randomly drawn from our pool of participants, using their item ratings and their (fitted) parameters. Then we applied the same exact BMC procedure that was used in the manuscript to analyze real choice data, with the same model space from H0 to H2.1. This model space includes the models that are the closest to each other (H1, H2 and H2.1) and hence the most difficult to distinguish. Indeed, they all predict a negative link between best-option choice rate and its serial position, albeit with a different shape. The recovery rate was perfect (100%) for models like H3 which predict instead a positive link. Recovery was not that perfect, however, for the critical models.

The results are provided in the confusion matrix (see Fig. S4 below), where cells indicate the proportion of cases in which the considered model in rows wins the comparison (has the highest expected frequency) when the considered model in columns was simulated. Recovery rate (in diagonal cells) was good for all models except H1, which was confused with H0. This is not surprising, given that in our participants, the bias parameter (λ) was often close to zero, making simulations of H1 close to those of H0, which wins the comparison because it saves one (useless) parameter. Importantly, when the winner was H2.1 (as with real data), the simulated model was either H2 or H2.1 in 100% of cases. This means that we can safely conclude that the covert pairwise comparison process (which is common to H2 and H2.1) was the most likely explanation of our dataset.

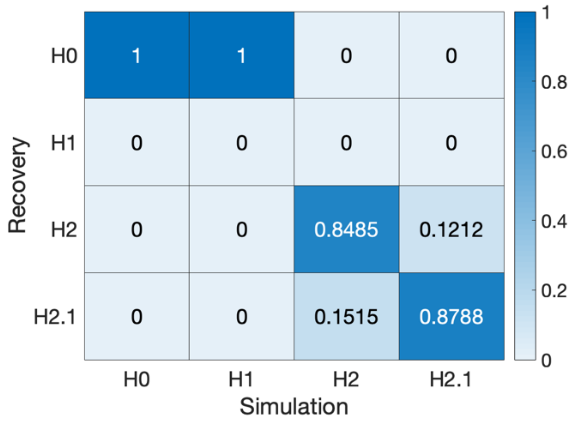

Fig. S4 – Model recovery analysis (Exp 1).
Choice data have been simulated using the likeability ratings and the posterior parameters of participants in Exp 1. Recovery rate has been established on the basis of 50 simulations, each including a group of 30 random participants. Cells of the confusion matrix indicate the rate at which the model in row wins Bayesian comparison when the model in column was simulated. Bayesian comparison was applied to simulated data at the group level, between the four considered models, following the exact same procedure applied to observed data. Only winning models with

exceedance probability > 0.95 have been included in the count. Note that when H2.1 model wins the comparison, the simulated model is either H2 or H2.1.

This figure has been included in the manuscript as supplementary information. The model recovery analysis is also mentioned in the main text (pages 22 - 23), as follows:

"To examine whether these models can be distinguished through Bayesian comparison, we conducted a model recovery analysis. Obviously, recovery success depends on computational parameters, since models are nested. For instance, model H1 with a null λ parameter is nothing but H0, and model H2.1 with a null δ parameter is nothing but H1. What we intended to check is whether a winning model, with an exceedance probability over 0.95, could be confused with another in the model space, given the observed choice data. We therefore used the observed likeability ratings, and computational parameters fitted on our choice data, to simulate virtual groups of participants. Bayesian model comparison was then conducted on simulated data in the exact same manner as with observed data. Recovery rate was good (see Fig. S4), except that H0 was winning when H1 was simulated. This is likely related to the fact that the bias parameter λ of model H1 was too small in our participants, making this model similar to model H0, which has the advantage of having one parameter less. Note that the impact of λ diminishes with the number of items, while the impact of δ is cumulative. The critical point was that when H2.1 was winning, the simulated model was H2.1 in a majority of cases and H2 in the others, thus validating the hypothesis of a covert pairwise comparison process."

Minor issues:

Page 8, Stimuli and apparatus & Page 15, Statistical analyses: Please include the version of Matlab used for data analysis and stimuli presentation.

→ We used Matlab_R2017a for stimuli presentation, and Matlab_R2018b for data analysis. This information has been incorporated into the revised methods (pages 8 and 16):

"All experimental stimuli were presented with Matlab_R2017a (https://www.mathworks.com/) running Psychophysics Toolbox-3 (Brainard, 1997) (http://psychtoolbox.org) and additional custom scripts."

"All analyses were run with Matlab_R2018b (www.mathworks.com)."

Page 8, Behavioral tasks: Please add information on the number of trials in description of Experiments 1 and 3.

→ This information was fully provided in Figure 1 (see below). It was also mentioned in the methods (pages 9-10). We now repeat the numbers, to make sure the reader would not miss the information (72 trials for Exp 1, 84 trials for both Exp 2 and 3).

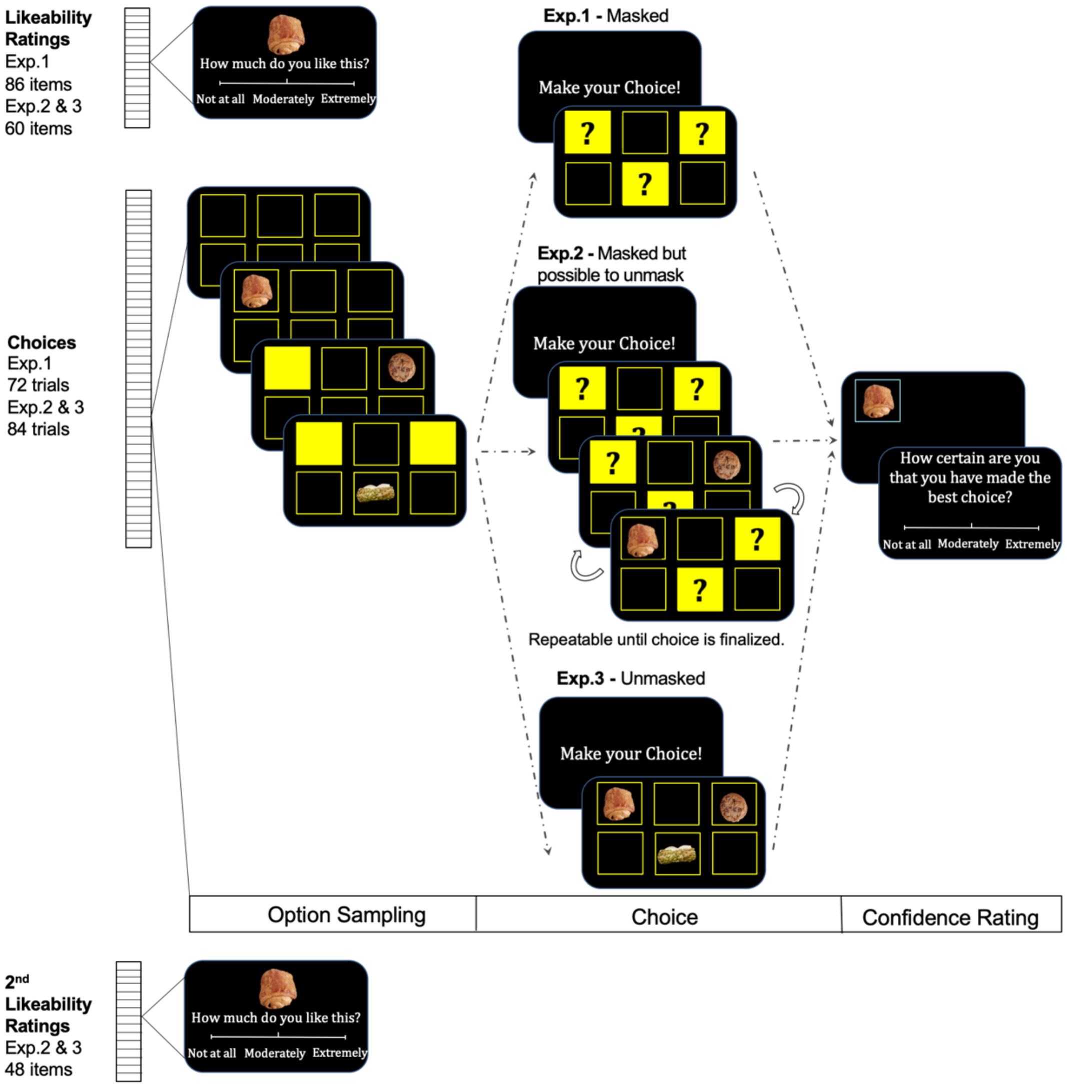

Fig. 1 - Behavioral tasks.
Each task session used one category of items (food, music, film or magazine) and was divided into two stages. In the first stage, participants had to rate the likeability of items presented one by one on the screen, by placing a cursor on a visual analog scale. In the second stage, participants had to browse through a set of 3 to 6 options, by pressing a bar on the keyboard, then click on their favorite option at the end of the sequence, and last, rate their confidence in their choice by placing a cursor on another visual scale. An additional third stage was included in Exp 2 and 3, with a second likeability rating task identical to the first one (presenting the same items). Beyond details regarding the number of items and categories, key differences between experiments regarded the choice phase: after being sampled, options remained masked (with no possibility of unmasking) in Exp 1, remained unmasked (with a time consuming possibility of resampling) in Exp 2, and were simply unmasked all together in Exp 3. Option sampling was self-paced, participants proceeding to the next option by pressing the space bar. At each step, a new option was revealed while the

previous one was masked again. The location of the options appearing on screen was randomized but their identity was prearranged on the basis of likeability ratings (see methods). Choice was prompted by displaying question marks on masks (Exp 1 and 2) or by unmasking all options together (Exp 3). A feedback showing the chosen option alone was provided before the confidence rating.

Page 10, third paragraph: … the persistence of choice-induced effects on subjective value. value updating. Please remove “value updating” at the end of the paragraph.

→ We thank the Reviewer for spotting this unfortunate repetition, which has been removed.

Page 15, Figure 2: B) “Shaded areas indicate the average S.E.M. across all datasets.” No shaded area is visible, please fix this issue.

→ We apologized for this incident, which happened during the automatic conversion to PDF format. We have made sure this would not occur when submitting the revised version.

Page 16, Results:
- Please add effect size analysis to your results section.

→ We now report mean and standard error for all regression coefficients and computational parameters whose significance was tested and reported in the results.

- Please add a table with mean and std of the extracted parameters for different models.

→ We now provide this table (below) in supplementary information.

Table S1. Fitted computational parameters (posterior mean and SEM)

Model	Parameter 1 (mean ± SEM)			Parameter 2 (mean ± SEM)		
	Exp 1	Exp 2	Exp 3	Exp 1	Exp 2	Exp 3
H0 (β)	0.086 ± 0.0071	0.105 ± 0.0079	0.093 ± 0.010			
H1 (β, λ)	0.086 ± 0.0072	0.106 ± 0.0079	0.093 ± 0.010	0.40 ± 0.056	0.47 ± 0.082	0.28 ± 0.043
H2 (β, δ)	0.080 ± 0.0063	0.103 ± 0.0089	0.093 ± 0.0098	0.61 ± 0.090	0.27 ± 0.098	0.037 ± 0.079
H2.1 (β, δ)	0.081 ± 0.0063	0.103 ± 0.0091	0.093 ± 0.0098	0.59 ± 0.089	0.29 ± 0.11	0.013 ± 0.087
H3 (β)	0.077 ± 0.0070	0.084 ± 0.0069	0.074 ± 0.0089			

This new Table is referred to in the revised results section (page 24):

"All fitted parameters are listed in Table S1."

Page 17, Figure 3:
- Please fix titles, legends, x/y labels.

→ Again this was due to an incident that happened during the automatic conversion to PDF format. We have restored the original and made sure it was correctly uploaded during submission.

- A) Are these probabilities calculated over all subjects? Can authors show a similar figure with average and S.E.M of these probabilities calculated separately for each subject?

→ The probabilities were in fact calculated separately for each subject, and then averaged across participants. This is what we meant by "inter-participant mean and standard error".

It is explained in the methods and repeated in the results (page 17):
"To assess the qualitative predictions of our computational model, we tested the relationship between P(best), the probability of choosing the best option (defined as the option with highest initial rating), and the serial position of that best option. The linear regression was done separately for each trial length (number of options) in each participant. Regression coefficients were then averaged, to obtain one value per individual and experiment, and tested at the group level (Fig. 3A)."

- B) "Shaded areas indicate the average S.E.M. across all datasets." No shaded area is visible, please fix this issue.

→ This was again due to the incident that happened during the automatic conversion to PDF format. We have restored the original and made sure it was correctly uploaded during submission.

Page 20, Figure 4: While H2.1 seems to be most likely model to explain the data in Exp. 1, the Exceedance probability of this model does not surpass chance level. Please comment on this.

→ The Reviewer surely means that exceedance probability was well beyond chance level, but failed to surpass the threshold of classical frequentist statistics (5% of false positives). This is correct: the exceedance probability of H2.1 in Exp 1 was 0.94, as acknowledged in the results section. However, we do not think this is a threat to the conclusion because

the second-best model is H2, which also implements covert pairwise comparison. If we sum the two models that implement covert pairwise comparison (H2+H2.1), the exceedance probability surpasses 0.99. Similarly, if we pool Exp 1 and Exp 2, the exceedance probability of H2.1 alone surpasses 0.99. We have added a comment on this point in the revised results section (page 22):

"The results of group-level random-effect Bayesian model selection are reported separately for the three experiments (Fig. 4). Model H2.1 (bonus + primacy model) was identified as the best model in Exp 1 (Ef = 0.63, Ep = 0.94) and Exp 2 (Ef = 0.76, Ep > 0.99). Although exceedance probability did not reach 0.95 in Exp 1, we note that its only competitor with non-zero exceedance probability was H2, meaning that in any case the hypothesis of covert pairwise comparison was the most plausible."

Page 20 & 21: Please mention what the value after the plus/minus symbols (±) refers to (e.g., ±std).

→ Throughout the manuscript, all intervals around means are standard errors, which correspond to the statistical tests employed (Student's t-tests). This is explicitly said in the methods (page 16), and reminded in the results section (page 20).

"Effect sizes are provided as means and standard errors of the mean (SEM)."

Page 28, Figure S1:
- Please fix titles, legends, x/y-axis labels.

→ This was again due to the incident that happened during the automatic conversion to PDF format. We have restored the original and made sure it was correctly uploaded during submission.

- Please add information on the modeled hypothesis to each panel.

→ Each panel is labeled with the model hypothesis (see below).

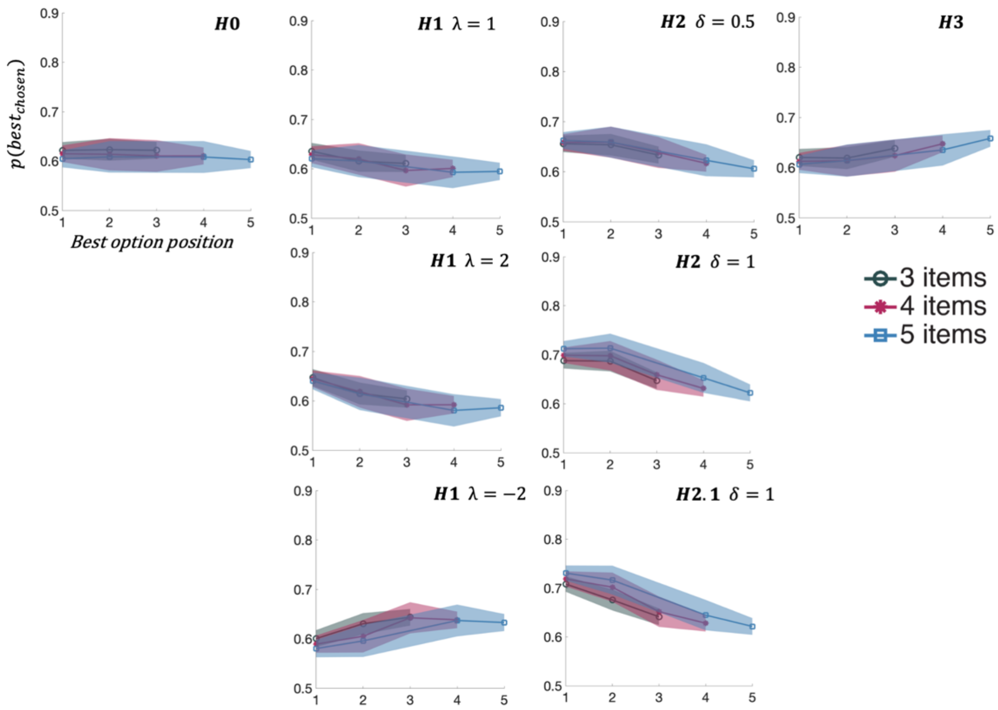

Fig. S1 - Simulations of choice data (under the settings of Exp 2 & 3).
Graphs show simulated probability of choosing the best option under the experimental setup of Exp 2 and 3, depending on the serial position of the best option in the sampling sequence (x-axis). Each of the plots is an average over 200 simulated datasets of 30 subjects implementing the corresponding model, for various (color-coded) number of options. Shaded areas indicate the average inter-participant SEM, across all datasets. Values of λ and δ are indicated on the plots. The inverse temperature parameter was fixed to $\beta = 0.10$, which corresponds to the posterior estimates of the best model (H2.1) fitted to choice data in Exp 2. The simulations show that predictions about the link between P(best) and its serial position are similar to those made under Exp 1 settings. In particular, only H1 (with a positive bias) and H2 (including H2.1) predict a decreased choice rate when the best option is presented later in the sequence.

- “Shaded areas indicate the average S.E.M.” No shaded area is visible, please fix this issue.

→ This was again due to the incident that happened during the automatic conversion to PDF format. We have restored the original and made sure it was correctly uploaded during submission.

Page 29, Figure S2:
- “Shaded areas indicate the average S.E.M.” No shaded area is visible, please fix this issue.

→ This was again due to the incident that happened during the automatic conversion to PDF format. We have restored the original and made sure it was correctly uploaded during submission.

- x-axis labels are missing.

→ This was again due to the incident that happened during the automatic conversion to PDF format. We have restored the original and made sure it was correctly uploaded during submission.

- Please add panel labels to the figure instead of using location (left/right) to refer to panels.

→ We have replaced left/right by A/B panels (previous Fig. S2 is now Fig. S3, see below).

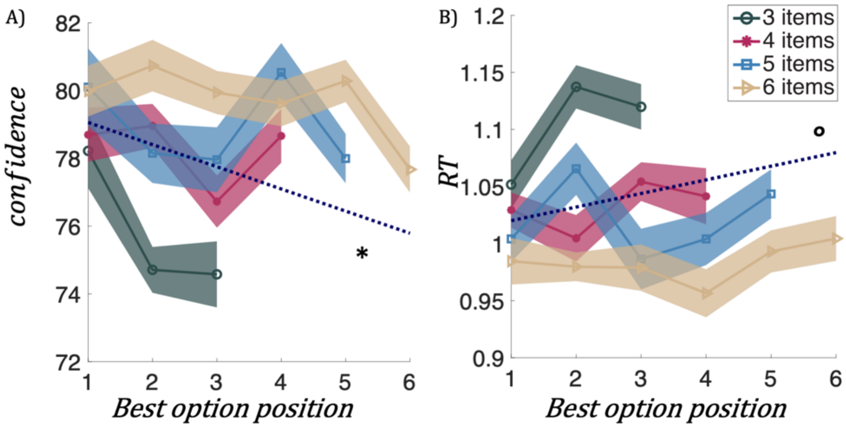

Fig. S3 – Model-free results about confidence and response time (Exp 1).
Graphs show the observed confidence rating (A) and response time (B), as a function of the serial position of the best option (in Exp 1), for different (color-coded) number of options, averaged over all trials. Shaded areas indicate inter-participant SEM. Dotted lines show linear regression fit across all trials (with different numbers of options). Star and circle denote significance or borderline significance of t-test comparing regression slopes to zero: * $p<0.05$, ° $p<0.1$.
